# A systematic review of the impacts of post-harvest handling on provitamin A, iron and zinc retention in seven biofortified crops

**DOI:** 10.1038/s43016-023-00874-y

**Published:** 2023-11-09

**Authors:** Samantha L. Huey, Elsa M. Konieczynski, Neel H. Mehta, Jesse T. Krisher, Arini Bhargava, Valerie M. Friesen, Mduduzi N. N. Mbuya, Eva C. Monterrosa, Annette M. Nyangaresi, Saurabh Mehta

**Affiliations:** 1 Center for Precision Nutrition and Health, Cornell University, Ithaca, NY USA; 2 Program in International Nutrition, Cornell University, Ithaca, NY USA; 3 Division of Nutritional Sciences, Cornell University, Ithaca, NY USA; 4 Global Alliance for Improved Nutrition, Geneva, Switzerland; 5 Global Alliance for Improved Nutrition, Washington, DC USA; 6 Global Alliance for Improved Nutrition, Nairobi, Kenya

**Keywords:** Developing world, Plant sciences, Agriculture

## Abstract

Post-harvest handling can affect micronutrient retention in biofortified crops through to the point of consumption. Here we conduct a systematic review identifying 67 articles examining the retention of micronutrients in conventionally bred biofortified maize, orange sweet potato, cassava, pearl millet, rice, beans and wheat. Provitamin A crops maintain high amounts compared with non-biofortified counterparts. Iron and zinc crops have more variability in micronutrient retention dependent on processing method; for maximum iron and zinc content, whole grain product consumption such as whole wheat flour or only slightly milled brown rice is beneficial. We offer preliminary suggestions for households, regulatory bodies and programme implementers to increase consumer awareness on best practices for preparing crops to maximize micronutrient content, while highlighting gaps in the literature. Our online, interactive Micronutrient Retention Dashboard (https://www.cpnh.cornell.edu/mn-retention-db) offers an at-a-glance view of the compiled minimum and maximum retention found, organized by processing method.

## Abstract (web summary)

Storage and processing can impact micronutrient levels of biofortified foods at the point of consumption. A systematic review and online dashboard present data on how processing methods affect micronutrient retention.

## Main

Approximately one in two women and children across the world continue to be affected by micronutrient deficiencies^1^. Whereas many populations, particularly those in low- to middle-income countries, are at risk of micronutrient deficiencies, women of reproductive age and children below the age of five years are the most affected. Diets in these countries largely rely on staple crops that are mostly energy dense but low in micronutrients. The situation is further aggravated by a lack of dietary diversity and/or affordable access to more nutrient-dense foods.

Biofortification typically focuses on staple crops and is the result of conventional selective plant breeding, agronomic management and/or genetic engineering techniques. Most biofortified crops have targeted an increase in provitamin A, iron and zinc concentrations, and their consumption has the potential to improve micronutrient intake and contribute to addressing micronutrient deficiencies globally. While the baseline nutrient levels of these crops are higher in their raw forms compared with their non-biofortified counterparts, there is evidence that post-harvest handling (PHH), storage, processing and shelf life and cooking/preparation methods can influence the retention of micronutrients in the foods. For example, it is known that storage and cooking can affect the concentration of some vitamins more than others due to oxidation and heat, and milling can result in mineral losses due to removing part of the husk and germ^2–6^. The evidence on micronutrient retention for biofortified crops has been discussed previously^7–11^ but not systematically reviewed.

The objective of this systematic review is to examine micronutrient retention after PHH in conventionally bred biofortified crops in varied-use settings, including (1) after storage of fresh and/or processed biofortified crops; and (2) after processing, such as milling or cooking.

## Results

**Fig. 1:**
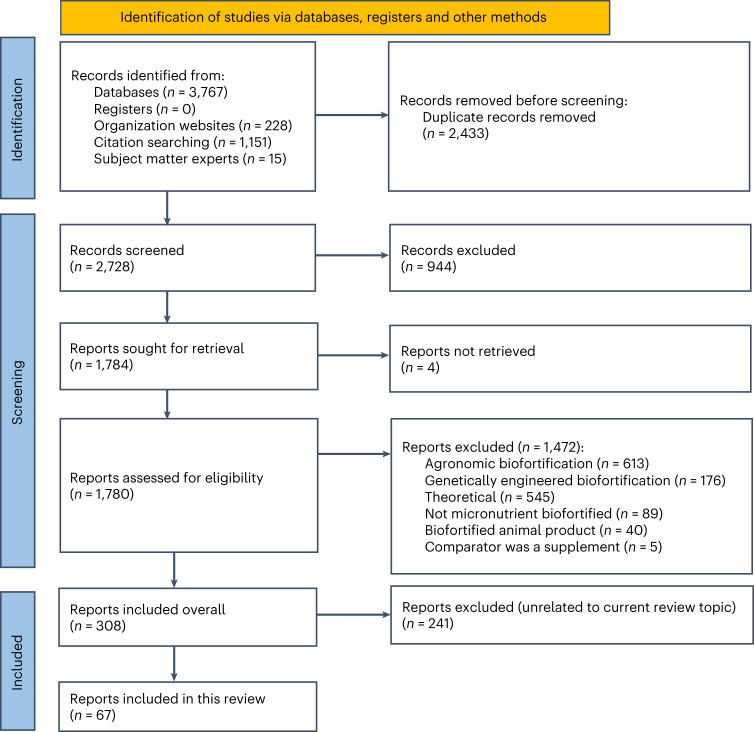
PRISMA Diagram for selection of studies included in this review. PRISMA Diagram^66^ shows the selection of 67 studies found under all four review topics.

For all four review topics (Supplementary Methods), we found a total of 5,161 records (Fig. 1). Ultimately, we identified 308 records as eligible for one or more of the four review topics outlined previously.

For this review, we identified 67 studies on micronutrient retention in conventionally bred biofortified crops. Across the review, ‘retention’ refers to ‘apparent retention’ unless otherwise noted as ‘true’ retention. For provitamin A-biofortified crops, provitamin A (PVA) content and beta-carotene equivalents (BCE) are described below.

We did not include results on micronutrient retention after storage of fresh mineral-biofortified crops, as losses are not expected.

Compiled results may be found in our online searchable Micronutrient Retention Dashboard (https://www.cpnh.cornell.edu/mn-retention-db), associated with this manuscript, and in Supplementary Table 1.

### Maize

We found 19 studies analysing PVA retention in maize (Supplementary Table 1), including one study that analysed zinc retention in zinc-biofortified maize.

Processing methods for maize included mashing, fermenting, roasting, microwaving, pressure cooking, boiling, storing, drying, extruding, frying, deep frying and milling into flour (Supplementary Table 2).

#### Fresh storage and PVA or BCE or zinc

Storing several unprocessed raw varieties of maize shelled into kernels or on the ears resulted in 40% BCE retention after six months’ storage, with the majority of degradation occurring in the first 15 days (ref. ^12^), following the first-order model of decay kinetics^13^. However, initially storing kernels at 4 °C for several days before storage at −20 °C resulted in higher retention of >100% (ref. ^14^); this 4 °C may have preconditioned the kernels, preventing oxidation and nutrient loss. A study examining storage of minimally processed (dehusked, sanitized) ears of BRS4104 maize for nine days in different types of packaging suggests vacuum sealing may be useful for short-term storage of this cultivar^15^.

#### Processing and PVA or BCE

From several studies^12,16–21^, processing unfermented maize by either cooking methods or grinding did not have negative impact on PVA or BCE retention. Variety appeared to impact PVA or BCE retention more distinctly, particularly for either unfermented or fermented maize. Retention of over 100% depended on processing method and appeared related to isomerization of beta-carotene and breakdown of the maize kernel and release of additional carotenoids. Boiling or drying maize resulted in high (~100% or greater) retention of zinc^17^.

#### Processing and storage and PVA or BCE

Studies found that aluminium packaging for longer-term storage of milled maize or dried maize kernels may be recommended^22,23^. These results also demonstrate the importance of the variety itself and type of packaging for long-term storage and suggest that adding an oxygen scavenger to minimize oxygen content may be ideal to minimize degradation during post-harvest storage^22^. It appears that generally, BCE content in cooked maize food products made from kernels that were stored for 90 days remained high^12,24^.

### Orange sweet potato

We found 28 studies analysing PVA retention in orange sweet potato (OSP; Supplementary Table 1). No studies measured PVA or BCE in OSP. Therefore, we summarize beta-carotene (BC) and all-trans beta-carotene (ATBC) retention.

Processing methods for maize included drying, chipping, storing, boiling, steaming, deep frying, roasting, pureeing, flaking and milling into flour (Supplementary Table 3).

#### Fresh storage and ATBC or BC

Storage for 15 days will reduce BC content by about 10% or more but depends on variety^25^.

#### Processing and ATBC or BC

Drying methods retained at least 60% of ATBC or BC but varied by variety^10,26–29^; the highest retention (99%) was found for ATBC after solar drying the Ejumula OSP variety^29^.

#### Processing and storage and ATBC or BC

Studies showed that packaging types along with other variables such as temperature, oxygen and light levels are critical to consider to maximize BC retention during storage in addition to the processing, such as boiling^25,30^, done on OSP before storage^31–33^. Whereas temperature data were not available from all studies, deep freezing at −80 °C was favourable for storing cooked OSP. Including materials to prevent both water vapour and oxygen from entering packaging for OSP flour appear to also be key in improving micronutrient retention.

### Cassava

We found ten studies analysing PVA retention in cassava (Supplementary Table 1). Only two studies measured BCE retention, which we summarize below. We also focused the results below on end products more likely eaten, including boiled whole cassava and porridge-like foods, and not intermediate steps (for example, grated and fermented cassava before cooking gari) for making a dish. Intermediate products and their micronutrient retention can be found in Supplementary Table 1 and in our Dashboard.

Processing methods for maize included fermenting, boiling, frying, drying, mashing and storing (Supplementary Table 4).

#### Processing and BCE

Processing method impacted retention values but variety and baseline amount of BCE determined the absolute amount of BCE after processing into food products^34–36^. Boiled whole cassava retained the most BCE content compared with porridge-like foods. *Chikwangue* and then fufu retained the least BCE content, which may be due to sieving for *chikwangue* or to the drying process during cossette formation for fufu^36^.

### Pearl millet

We found four studies analysing iron and iron retention in pearl millet (Supplementary Table 1).

As seen in Supplementary Table 5, processing methods for pearl millet included soaking, germinating, decorticating, refrying, malting, roasting, fermenting and milling into flour.

#### Processing and iron or zinc

Parboiling and oven drying biofortified pearl millet may be advantageous for higher iron retention^37^. If soaking is preferred, soaking pearl millet in a grain:water ratio of 1:5 for 12 hours may maximize retention^38^. Soaking may be advantageous to allow fermentation^37^ and lactic acid bacteria phytase activity to break down phytates, increasing iron bioavailability. Malting and germination^39^ were found to decrease iron retention in whole grains, but germination of raw flour maintained high iron retention. Iron contamination from cooking utensils should be considered in assessing iron retention^37,40^.

#### Processing and storage and iron or zinc

Iron retention was high (88% to ≥100%) after various processing steps (parboiling, oven drying, milling and/or steeping and fermenting) and storage thereafter for ≤1 month did not negatively impact iron retention^37^.

Similar to iron, parboiling and oven drying biofortified pearl millet may be advantageous for higher zinc retention. If soaking is preferred, soaking pearl millet in a grain:water ratio of 1:5 for 12 hours may maximize retention^38^. Malting and germination were found to decrease zinc retention of whole or decorticated pearl millet^37,40^, but germination of raw flour maintained high zinc retention.

Again similar to iron, zinc retention was nearly 100% after various forms of processing, and post-processing storage of ≤1 month maintained the zinc retention.

### Beans

We found three studies analysing iron and zinc retention in beans (Supplementary Table 1).

As seen in Supplementary Table 6, processing methods for beans included drying, milling, parboiling, steeping and polishing.

#### Processing and iron or zinc

Iron overall was well retained across a variety of bean-processing methods^41–47^. Boiling and processing into flour all resulted in retentions approaching or over 100%. Extrusion may be preferred over malting/roasting raw flour to enhance nutrient retention. Iron retention after refrying with iron-free cooking broth remains a research gap.

Overall, zinc was retained across a variety of bean-processing methods^41,42,44^. Boiling, refrying and processing into flour all resulted in retentions approaching or over 100%, depending on variety for milling. In the case of zinc, malting/roasting may be slightly preferable to extrusion, but both methods result in similarly high zinc retention.

### Rice

We found two studies analysing iron and zinc retention in rice (Supplementary Table 1).

Processing methods for rice mainly included cooking and polishing to various degrees of milling (Supplementary Table 7).

#### Processing and iron or zinc

Rice variety impacted the level of iron retention across the varieties and processing methods^48,49^; even the same rice variety varied depending on where it was grown, contributing another variable into determining micronutrient retention. However, polishing rice at 5%, 7.5% or 10% degrees of milling consistently reduced iron content by about 50%. Consuming brown rice without polishing will be most beneficial for maximal iron intake.

Similar to iron, rice variety impacted the level of zinc retention across the varieties and processing methods^48,49^. Rice grown in Santa Rosa appeared to consistently result in higher zinc content post-processing than rice grown in Palmira. Polishing at 5%, 7.5% or 10% degrees of milling consistently reduced zinc content by about 20–40%. Consuming brown rice without polishing will be most beneficial for maximal zinc intake.

### Wheat

#### Processing and zinc

Milling at 95% extraction is preferred to 80% extraction for zinc retention in biofortified wheat^50^.

## Discussion

This review sought to summarize the evidence on micronutrient retention in conventionally bred biofortified staple crops after post-harvest handling. PVA-biofortified crops were better represented across the identified studies compared to iron/zinc-biofortified crops. Variety impacted micronutrient retention within similar processing methods. Overall, more research is needed to better understand micronutrient retention in mineral-biofortified crops to ultimately guide adoption and scale up efforts globally.

### Comparison with non-biofortified conventional crops

Biofortified crops yield greater micronutrient levels compared with non-biofortified crops in general, even after storing or processing. For example, assuming adequate relative humidity, the lowest absolute PVA/BCE content in biofortified maize after boiling 30 minutes and frying in soybean oil was 1,145 µg 100 g^−1^ (suboptimal retention of 48%)—higher than the baseline PVA content in non-biofortified white maize. This finding of biofortified crops having higher micronutrient levels compared with non-biofortified crops was also consistently observed for OSP and yellow cassava.

For iron and zinc, using the recommended methods (parboiling, oven drying) for processing pearl millet, we find a minimum retention of 53 µg g^−1^ (representing full retention of 100%) iron and 40 µg g^−1^ zinc (high retention of 98%), compared with 20 µg g^−1^ iron and 19 µg g^−1^ zinc in conventional millet^51,52^. The lowest absolute iron and zinc concentrations retained after processing (refrying, boiling) whole beans was 82 µg g^−1^ (high retention of 98%) and 30 µg g^−1^ (high retention of 77%), respectively, while milling beans into flour resulted in 48 µg g^−1^ (high retention of 72%) iron and 22 µg g^−1^ (high retention of 92%) zinc, compared with non-biofortified beans containing ~55 µg g^−1^ iron and ~28 µg g^−1^ zinc^53,54^. However, a recent study showed no differences in iron and zinc concentrations in biofortified and non-biofortified beans in the East African marketplace^55^. Iron and zinc levels in processed (polished) biofortified rice were mostly at or above the levels of conventional polished rice ( ~ 2 µg g^−1^ iron and ~16 µg g^−1^ zinc)^48^ after polishing, though only dehulling to form brown rice would result in higher micronutrient content. Finally, non-biofortified wheat contains 24 mg g^−1^ zinc (moderate retention of 58%); as noted in our review, milling at 95% extraction is needed to produce processed wheat with higher zinc levels (40 µg/ g^−1^, via high retention of 98%). Only one study examined zinc-biofortified maize; retention of zinc content remained around 100% or greater after boiling and drying, which is expected given zinc is not degraded by heat.

Broadly, these results show that despite micronutrient losses during certain forms of processing, biofortified crops still retain higher amounts of micronutrients than non-biofortified crops before any form of processing. However, certain gaps in our knowledge on retention after different processing methods and by crop and variety remain, as outlined in the results.

### Comparison with fortified crops

In contrast to biofortification—which is carried out through either conventional plant cross-breeding, agronomic methods or genetic engineering to achieve higher micronutrient content inherently in the crop, before processing—fortification is the practice of increasing the content of micronutrient(s) in a food or condiment to improve its nutrition content during or after processing^56^. This includes both adding additional vitamins, minerals or other trace elements to a food, and adding back in the micronutrients that were lost during processing, such as replacing iron, folic acid, niacin, riboflavin and thiamine micronutrients that are lost during milling of wheat flour (also known as enrichment)^56^. Because fortification occurs during or after processing, it is not always possible to compare micronutrient retention values because many of the biofortified foods include whole boiled sweet potato or cassava and so on without additional milling or other processing. Also, with fortification, it is possible to add other micronutrients not inherently present in a food product, such as vitamin A in wheat flour; biofortified crops-based foods target only the micronutrients that are already found in the crop. The stability of vitamins remains a limiting factor in the success of fortification programmes in regard to flour, given that flours are usually not consumed immediately and instead stored for several months^57^. Depending on storage conditions including type of packaging, duration, presence of other micronutrients and temperature, a vitamin A retention as high as 95% to as low as 30% has been observed^57^. Other fortified foods such as vitamin A-, iron-, zinc-, folic acid- and vitamin B12-fortified rice showed high retention values of 75–100% overall except for vitamin A after hot extrusion, cold extrusion and coating^58^.

In our review, one study found that PVA-biofortified yellow maize flour (average of three varieties: ~2,397 µg beta-carotene 100 g^−1^ flour) made into porridge yielded 78–99% (high) retention of beta-carotene^59^, the precursor to vitamin A; in comparison, white maize flour fortified with vitamin A (261 µg retinol activity equivalent (RAE) 100 g^−1^) yielded a retention value of 40% (suboptimal retention) in one study^60^. Considering that 1 µg RAE = 2 µg beta-carotene, we are left with about 936–1,200 µg RAE in the biofortified porridge and about 104 µg RAE 100 g^−1^. However, as discussed earlier, this depends greatly on biofortified genotype and exact processing steps used (such as including fermentation or not). A recent review discusses processing-related vitamin losses in fortified and biofortified cereals^61^; however, comparing micronutrient retention in terms of minerals and between other biofortified and fortified foods remains a research gap.

### Limitations of this review

The level of contamination particularly for mineral-biofortified crops was not mentioned in some studies. Aluminium concentration over 5 µg g^−1^ dry weight is considered an indication of possible Fe contamination^55^. Extra iron or zinc from other recipe ingredients, cookware or the cooking water may inflate the iron and zinc retention values here. We highlighted in each study where contamination was addressed in the Supplementary Information and Results. Further, there may be additional variability based on differences in laboratories and methods used across different studies to measure micronutrient concentration across the range of foods included in the review.

Measuring the bioaccessibility and bioavailability of the micronutrients retained after processing needs to be routinely assessed as it is critical to truly understand how beneficial each crop, process and food product will be to improving micronutrient status in populations. The bioaccessibility and bioavailability of micronutrients in biofortified crops have been examined in a recent review^62^.

### Future directions

On the household level, the processes that maximized micronutrient retention included: boiling or roasting maize with a lid or in the husk, respectively; drying unpeeled OSP; boiling whole cassava, parboiling or oven drying pearl millet, boiling beans with or without refrying, dehulling rice without polishing. There was only one study on biofortified wheat, constraining our ability to make a recommendation for wheat processing.

On the national level, as recommended by the National Biofortification Guidelines of Tanzania^63^, regulatory bodies or technical institutions should ensure that manufacturers of processed biofortified food products include instructions clearly showing how the product should be prepared to retain maximum micronutrient content, particularly for PVA-containing biofortified food products. Further, it is important to account for micronutrient retention across the biofortified crops value chain, which involves a range of actors, including processors, retailers and consumers. Depending on the desired food product, processors should consider genotypes that contain high baseline micronutrient content and are more amenable to particular processing and storage techniques to maximize micronutrient retention in the final product. For these, flour extraction rates are also subject to consumer taste; even if brown rice retains the maximum iron and zinc, processors and retailers will not be able to sell this to consumers who prefer polished rice, ultimately not improving micronutrient status.

Additionally, accounting for geography and setting will be crucial to the success of biofortification, particularly for beans. A recent review on biofortified beans outlined three major assumptions for the biofortification approach^64^: first is how much nutrient content is actually in non-biofortified beans; second is whether iron-biofortified beans have accompanying higher bioavailability; and third is that such beans can be bred using traditional methods and are sustainable. In the case of beans, non-biofortified beans appear to have higher iron content than previously thought; iron-biofortified beans do not necessarily have more bioavailable iron; and as noted in Colombia with rice, there is a strong genotype by environment interaction, leading to varying levels of micronutrients even within the same variety. Finally, given that biofortification is a relatively new nutrition intervention, it is important to include education on micronutrient retention after processing or storing crops and food products for processors and retailers in addition to the educational materials that are part of general awareness-generating campaigns among consumers.

## Conclusions

Overall, PVA crops maintain high amounts of PVA compared to non-biofortified counterparts. Iron and zinc crops have more variability in micronutrient retention dependent on processing method, and for maximum iron and zinc content, it would be helpful to consume whole grain products such as whole wheat flour or only slightly milled brown rice. There remains a gap in the literature on storage and shelf life across PVA crops, and ideal temperature, humidity and maximum durations are important considerations for retailers of biofortified food products. Finally, including bioaccessibility and bioavailability data in studies examining micronutrient retention are needed.

## Methods

We registered the protocol for this review on PROSPERO (CRD42021254461) on 11 June 2021^65^. Methods are detailed in the protocol and in the Supplementary Information and are briefly described below.

### Inclusion and exclusion criteria

We included studies examining biofortified foods and food products, including those that have undergone PHH, including storage of fresh crops, processing (for example, drying, milling, grinding, cooking, freezing) and post-processing storage/shelf life, that have been delivered as crops only or in the form of food products. Crops included those biofortified by conventional plant breeding approaches. We did not include interventions using agronomic nor genetic engineering-based biofortification methods or animal-based biofortified foods such as dairy products or meat from animals that consumed biofortified feed. Comparisons were different varieties of different biofortified crops.

### Outcomes

The primary outcome was micronutrient retention (apparent retention and true retention) in biofortified crops and the impact of factors such as storage of fresh or raw crops, processing and post-processing storage/shelf life. Definitions and methods to measure micronutrient retention are described in our Supplementary Information.

### Study designs

We included any studies that measured micronutrient content of biofortified crops before and after some form of processing. Studies that modelled, predicted or estimated how processing of biofortified crops may impact our outcomes of interest were excluded.

### Literature searches

We originally aimed to conduct a set of four reviews on biofortification and thus designed our search strategy to accommodate the topics examined by all four reviews (original protocol^65^).

We performed a search of relevant literature databases including: MEDLINE (PubMed), AGRICOLA, AgEcon, CABI Abstracts (Web of Science) and organizational websites (for example, HarvestPlus, CGIAR and partners).

These, including the original MEDLINE search, are summarized in Supplementary Table 8. We also hand searched organization websites. The results are included in Supplementary Table 9.

We also identified 1,151 potential citations outside of the original search during the screening process. These included studies that were: cited in review papers but did not include variations of the term ‘biofortification’ in their abstracts; not indexed in any of the literature databases described above and were thus missed by the original search; published recently in 2021, which we identified from journals’ table of contents alert feeds. Some of the latter included full-text versions of conference abstracts that were found and included in the original screening pool. We used EndNote X9 software for citation management.

### Data screening and extraction

All records were screened for eligibility at the title/abstract level and then at the full-text screening level. We used Covidence (www.covidence.org) to screen and organize studies.

Data were extracted for each identified study using Microsoft Excel 16.77.1 and FileMaker Pro 19 and PlotDigitizer software (https://plotdigitizer.sourceforge.net/).

### Data synthesis and analysis

We back calculated micronutrient retention outcomes measured as losses (%) to their reciprocal to condense outcomes and increase consistency across the review. These are being considered ‘retention values informed by losses’ and are included as apparent retention.

We calculated apparent retention if articles reported only the micronutrient concentration of interest per unit of dry weight before and after processing to arrive at approximate retention values. We also back calculated the absolute micronutrient concentration if enough information was available.

In addition to the retention of individual carotenoids and total carotenoid content including those with and without provitamin A activity, such as zeaxanthin, lutein or lycopene, detailed tables for micronutrient retention of each crop with separated outcomes for apparent retention, true retention and losses are available in Supplementary Table 1.

From our review of the literature, we have adapted interpretations of the retention values we report in this review, as follows:

>70%: high retention

50–70%: moderate retention

<50%: suboptimal retention

We note that there is no consensus on these scales but have proposed these for easier interpretability of our findings.

### Reporting summary

Further information on research design is available in the Nature Portfolio Reporting Summary linked to this article.

### Supplementary information

Supplementary Information Supplementary Methods, Results, Tables 2–9, Discussion and References.

Reporting Summary

Supplementary Table 1 All extracted retention data from individual studies’ experiments.

## Data Availability

All data generated or analysed during this study are available in the Supplementary Data of this article and in our Micronutrient Retention Dashboard: https://www.cpnh.cornell.edu/mn-retention-db. The following databases were used in this study: MEDLINE via PubMed (https://pubmed.ncbi.nlm.nih.gov), AgEcon (https://ageconsearch.umn.edu), AGRICOLA (https://web-p-ebscohost-com.proxy.library.cornell.edu/ehost/search/advanced?vid=0&sid=0ee440e5-8df0-48e8-be66-245addd56794%40redis) and CAB Abstracts (https://www-cabidigitallibrary-org.proxy.library.cornell.edu/product/ca).
