# Supplementary material for: A systematic review of the impacts of post-harvest handling on provitamin A, iron and zinc retention in seven biofortified crops
Source: Nat Food. 2023 Nov 9;4(11):978–85. doi: 10.1038/s43016-023-00874-y (PMC10661739; doi:10.1038/s43016-023-00874-y)
Supplement: Supplementary file 1 — Supplementary Methods, Results, Tables 2–9, Discussion and References. [file 43016_2023_874_MOESM1_ESM.pdf]

nature food

Article https://doi.org/10.1038/s43016-023-00874-y

A systematic review of the impacts of post-harvest handling on provitamin A, iron and zinc retention in seven biofortified crops

In the format provided by the authors and unedited

Contents:

Methods: additional details 3
Results: additional details 4
  Maize 4
  Orange sweet potato 6
  Cassava 9
  Pearl millet 10
  Beans 11
  Rice 12
  Wheat 14
Supplementary Tables 15
  Supplementary Table 2. Characteristics of included studies: maize 16
  Supplementary Table 3. Characteristics of included studies: orange sweet potato 25
  Supplementary Table 4. Characteristics of included studies: cassava 35
  Supplementary Table 5. Characteristics of included studies: pearl millet 40
  Supplementary Table 6. Characteristics of included studies: beans 41
  Supplementary Table 7. Characteristics of included studies: rice 42
  Supplementary Table 8. Search strategy across included databases 44
  Supplementary Table 9. Results from hand-searching organization websites 45
Discussion: Additional points 46
Supplementary Information References 49

Abbreviations: AR, apparent retention; ATBC, all-trans beta-carotene; BC, beta-carotene; BCE, beta-carotene equivalents; PVA, provitamin A; TR, true retention

Keywords:
Biofortification
Biofortified food products
Micronutrient retention
Iron
Zinc
Vitamin A

Methods: additional details

Micronutrient retention. Background and methods. "Micronutrient retention" describes the amount of micronutrient content retained after processing or storage.

Apparent retention (AR) is the nutrient content of the moisture-free (dry) weight of processed foods relative to the food's raw form. AR is calculated as follows[1]:

$$\% AR = \frac{[nutrient\ content\ per\ g\ of\ processed\ food\ (dry\ weight)]}{[nutrient\ content\ per\ g\ of\ raw\ food\ (dry\ weight)]}$$

Unlike AR, **true retention (TR)** not only considers the amount of micronutrients before and after processing, but also resulting changes in the weight of the product[2]. TR is calculated as follows:

$$\% TR = \frac{[nutrient\ content\ per\ g\ of\ processed\ food *\ g\ of\ food\ after\ processing]}{[nutrient\ content\ per\ g\ of\ raw\ food *\ g\ of\ food\ before\ processing]}\ x\ 100$$

Losses, defined as the amount of micronutrient lost in a food after processing, is another method to measure micronutrient retention, and is the reciprocal of retention. It can be calculated using the following equation[3]:

$$\% Loss = 1 - [nutrient\ content\ per\ g\ of\ processed\ food - nutrient\ content\ per\ g\ of\ unprocessed\ food] \times 100$$

For PVA-biofortified crops, we summarized the results on retention of total PVA and/or BCE as defined by trialists. Both PVA and BCE relate to the amounts of carotenoids with provitamin A activity, which comprised ATBC, cis-beta-carotene isomers, alpha-carotene, beta-cryptoxanthin (BCX), and/or alpha-cryptoxanthin. If the retention of total PVA content or BCE was not reported for a given crop (e.g., OSP) we summarized results on the retention of total BC and/or ATBC.

Results: additional details

Maize

PVA or BCE: Fresh storage. Storing raw GV662A (HP1002), GV665A (HP1005), HP1001, and HP1003 shelled into kernels or on the ears harvested at 7 months in ambient conditions for 180 days resulted in about 40% BCE retention[4]. As expected by the first order model of decay of carotenoids during storage[5], the majority of BCE degradation occurred within the first 15 days (to 60% retention), followed by a further 10% reduction over the next 15 days and remaining relatively stable at ~50% until day 120, decreasing to 40% by day 180. In another study, storing the raw dehusked cob for genotype "2–9 x 11–7" for 5, 6, 9, 12, or 15 days at 4 ºC, and then an additional 3 months at -20 ºC, resulted in retention values >100%; the number of days in 4 ºC storage yielded the highest retention at 12 days (130%)[6]. The initial 4 ºC storage was hypothesized to have "pre-conditioned" the kernels to avoid detrimental effects of being stored directly at -20 ºC just after harvest, such as structural damage and leakage of cellular contents from damaged cells resulting in oxidation of carotenoids[6].

Another study examined PVA retention in minimally processed (dehusked, sanitized) whole ears of maize (BS41014) stored for 9 days, either in polystyrene trays covered with PVC film or in vacuum-packed trays in multilayered nylon, at 5 ºC[7]. At days 3, 6, and 9, the percentage retention increased (from 40% to 49% [1.33–1.63 µg/g) compared to raw cobs prior to storage) in the vacuum-sealed packaging. In polystyrene with PVC film packaging, retention was highest at day 6 (47%, 1.56 µg/g) and similar at days 3 and 9 (~36%, 1.19–1.20 µg/g). These results suggest vacuum sealing may be useful for short-term storage of whole BRS4104.

PVA or BCE: Processing. Raw BRS4104 cob in the milky stage that was microwaved without water resulted in 56% true retention of PVA (1.19 µg/g)[8].

Pressure cooking milky BRS4104 cobs in water, or boiling PVA-biofortified varieties in a pot with or without a lid resulted in 51% to 109% PVA true retention (1.37–2.58 µg/g)[8]. Boiling maize in a lidded pot may concentrate carotenoids that may be otherwise lost[8].

Boiling or roasting raw maize kernels (varieties HP1001, HP1002, HP1003, HP1005) resulted in similar micronutrient retention values and absolute BCE content[4]. Boiling (retention: 89–138%) resulted in about 9.19–10.35 µg/g DW BCE, while roasting (retention: 58.6–120%) resulted in about 6.04–9.83 µg/g DW BCE). In another study, a combination of 23 PVA-biofortified subtropical maize hybrids that were boiled resulted in 92.24% retention of PVA (6.42 ± 3.11 µg/g)[9]. After roasting, cis-isomers of beta-carotene increased among these genotypes, resulting in BCE retention >100%[4].

For whole maize cobs or kernels, it appears that boiling and roasting—which are common methods for maize preparation—may be most suitable for PVA and BCE retention. Microwaving may not be recommended, but there was a lack of data for this method.

Nixtamalization is the process of soaking and cooking in alkaline solution such as limewater to soften and prepare maize for grinding. Seven maize varietals (BEH1, BEH2, BEH3, BEH4, BEH5, BEH6, EH7) underwent nixtamalization using two methods: traditional and extrusion[10]. Flour itself, tortilla dough, and cooked tortillas varied by varietal in terms of BCE retention. BEH5 retained 97% of BCE in flour via traditional nixtamalization and was over 100% in tortilla dough and cooked tortillas (~7.82–7.90 µg/g DW). Extrusion resulted in slightly lower BCE retention. It

appears that traditional nixtamalization is suitable for BCE retention for making tortilla products, but varietal must be taken into account.

Raw kernels from HP1001, HP1002, and HP1003 as well as the three varietals combined harvested at 7 months' maturation that were hammer-milled into mealie meal retained BCE (81–110%, 6.46–11.33 µg/g DW)[4]. Raw kernels from 10 MAK 7-7 yielded retention of 137% PVA [10.6 µg/g DW] after cleaning, degerminating, and roller-milling into raw mealie meal[11]. The high retention observed may be due to increasing the amounts of available carotenoids due to breakdown of the maize kernel matrix[11].

Steeping and fermenting maize for 24–120 hours and subsequently freeze-drying and milling into flour resulted in BCE retention values ranging from 61–122% (2.7–6.6 µg/g) compared to the unfermented maize for varietals C17 × DE3; Hi27 × CML328; 2013 Orange ISO; 2015 Orange ISO; [KUI carotenoid syn-FS17-3-1-B-B-B-B-B-B-B-B] × [(MAS[206/312]-23-2-1-1-B-B-B/[BETASYN]BC1-6-5-1xFloridaASYN#-B)-B-1-3-B-B-B][12]. It appears that varietal (C17 × DE3) resulted in the highest BCE retention values (>100%) regardless of duration of fermentation. Boiling the fermented flour into ogi and storing at -80 ºC resulted in varying effects on retention as compared to unfermented ogi. A fermentation period of 24–72 hours resulted in higher BCE retention values than 120 hours fermentation, across all varieties, likely due to carotenoid leaching into liquids over time during fermentation.

Soaking mealie meal in cold water followed by decortication, creating raw samp, resulted in high BCE retention (96–123% or 7.68–11.12 µg/g DW)[4]. Washing and boiling the steeped, decorticated samp into cooked samp resulted in lower BCE retention values ranging between 53–98% (4.22–10.07 µg/g DW), with HP1005 resulting in the highest retention.

Boiling mealie meal into thin porridge also resulted in high BCE retention compared to raw kernels for all—106–127% or 9.21–11.35 µg/g DW—but HP1002, which decreased to 59% (4.72 µg/g). However, boiling and simmering mealie meal into nshima, a thick porridge, resulted in over 100% BCE retention (8.26–16.89 µg/g DW)[4]. Super maize meal, achieved by roller-milling, that was boiled into porridge retained over 100% of PVA (2.24–3.18 µg/g) compared to raw super maize meal[13].

PVA or BCE: Processing and Storage. After first drying raw OPVI and OPVII cobs to 8.5% moisture, or drying GV664A for 3 days in shade, the BCE retention depended on the specific packaging used[14]. Retention was lowest, at 48–51.4%, when GV664A kernels were stored for 6 months in a metal silo without a seal (3.80 µg/g) or in woven bags (3.90 µg/g), or kept on the cob and stored in woven bags (3.60 µg/g DW)[15]. In this study, all PVA followed first-order degradation kinetics[15]. Retention was highest when GV664A was stored in aluminum bags for any duration (96–104%, 7.36–8.00 µg/g DW). Freezer-milling or rotor-milling Pool 8A maize and storing for 10–180 days in various packaging (aluminum pouch, laminated paper bag, double-layered polyethylene bag) at either 4 ºC or 37 ºC resulted in differences in PVA retention[16]. At 180 days storage, the lowest PVA retention was observed for freezer-milling and storage in a laminated paper bag at 37 ºC, while the maximum retention (77%) was observed for rotor-milling and storing in an aluminum pouch at 4 ºC. BCE and PVA retention values after 8 months of storage were reported highest for dried OPVI and OPVII using Purdue Improved T Crop Storage (PICS) bags with an oxygen scavenger ("PICS-OXY") packaging (46%, 2.64 µg/g DW)[14]. Generally, OPVI yielded higher BCE and PVA retention than OPVII.

Vacuum oven-drying raw kernels of several varieties (2012 Orange ISO Selected A, (CI7×DE3)×2010-Orange-Isolation, CI7×DE3, Hi27×CML328) at 40 ºC for 48 hours, and subsequent storage for 4, 7, 27, or 53 weeks in 4, 22.5, 55 ºC, at RHs of 0.65%, 59%, 64% in a salt solution resulted in PVA retention values mostly below 50%, including as low as 0%[17]. Higher retention was observed in conditions with 64% RH (19–55.9% PVA retention, or 2.35–4.12 µg/g DW) compared to 0.65% RH (0–6.7% for all except [KUI carotenoid syn-FS17-3-1-B-B-B-B-B-B-B-B] × [(MAS[206/3])] which still yielded 48% retention[17]. In this study, PVA followed first-order degradation kinetics[17]. The results of this study show that carotenoid stability in maize kernels during storage was dependent on temperature, genotype, and crucially, moisture.

Cooking porridge, nshima, and samp made from kernels of varieties HP1001, HP1002, HP1003, HP1005 stored for 90 days did not appreciably alter BCE retention values[4]. For nshima, all BCE retention values were over 100% except for HP1003 upon storing which decreased to 67% or 5.02 µg/g). For porridge, all values were over 100% except for HP1001 and HP1003 after storage, while HP1002 increased from 58.9% BCE over 200% retained from the raw kernel content after 90 days). For samp made with grits that were stored for 90 days, all BCE retention values increased to over 100%.

Zinc: Processing. One high-zinc maize variety was examined in one study, which authors referred to as “high kernel zinc maize, 10 genotype combination”[9]. Boiling resulted in 116% retention of zinc, while drying resulted in nearly 100% retention of zinc. This is expected given that zinc (and other minerals) is not lost due to heat[18, 19].

Zinc: Processing and Storage. No studies examined processing with post-processing storage of any zinc-biofortified maize.

Orange sweet potato

ATBC or BC: Fresh storage. One study examined BC retention in 15 raw OSP varieties harvested at 120 days after pollination after a 15-day storage period[20]. BC true retention values ranged between 71–92% (2.21–8.69 mg/100 g), with variety 187017-1 having the highest retention but ST-14 yielding the largest absolute amount of BC. Variety S-1281 showed the lowest retention while variety 440127 yielded the absolute lowest amount of BC. From these results, storage for 15 days will reduce BC content by about 10% or more but depends on variety.

ATBC or BC: Processing. Raw, chipped or sliced OSP was dried using a variety of methods including hot air, solar-tunnel, direct sun, cross-flow, and oven. Drying is a preservation method for OSP consumption during the off-season[21].

After hot air drying chips from a United States-sourced OSP variety, Rubina® Agrexco Carmel Rungis, for 2 hours, 84% of ATBC (247 µg/g) was retained[3, 22].

Solar-drying chips of Rubina® Agrexco Carmel Rungis in a greenhouse resulted in 77% retention (226 µg/g)[3, 22]. Solar-drying peeled Ejumula in the open air for an unspecified amount of time resulted in 99% ATBC retention (264.6 µg/g DW)[21].

Sun-drying was examined across other OSP varieties, including the Rubina® variety[3, 23] as well as Ejumula[21], KS-7, ST-14-1, ST-14-16, ST-14-34, ST-14-49, ST-14-53, ST-14-6, ST-14-9, ST-3-17, ST-3-22[24]. Sun drying chipped Rubina® for 8 hours resulted in ATBC retention values

ranging between 66–68% (193–199 µg/g DW), while 6-10 hours drying time for sliced Ejumula resulted in 84% ATBC retention (252.7 µg/g DW). Retention across 10 different varieties [24] after sun-drying for 48 hours resulted in similar BC true and apparent retention values ranging between 66–73% (9–9.3 µg/g DW).

Cross-flow drying resulted in 79–84% ATBC retention of the Rubina variety (232–247 µg/g DW)[3, 22].

Oven-drying sliced Ejumula for 10 hours at 57ºC resulted in 88% retention of ATBC (274 µg/g DW)[21]. Oven drying sliced and blanched KS-7, ST-14-1, ST-14-16, ST-14-34, ST-14-49, ST-14-53, ST-14-6, ST-14-9, ST-3-17, ST-3-22 for 50–60 ºC for 24-48 hours resulted in BC retention of 82–96% (7–12.7 µg/g FW), which upon frying further reduced by 10–20% depending on varietal[24].

Several studies examined boiling of OSP, a common way to consume this crop[20, 21, 25, 26, 27, 28, 29, 30]. Generally, the longer OSP is boiled, the lower the BC retention; the highest true retention (97%) was found for peeled, cubed Kulfo and Tulla OSP and 99% for peeled Yanshu No. 5 OSP boiled for 10 minutes[26, 28]. However, for usual consumption, whole OSP must be boiled until internal temperature reaches 86 ºC; to achieve this level of cooking, the size of the potato informs the duration of boiling needed, which is generally at least 30 minutes. BC true retention in quartered Kulfo and Tulla was 57–83% after 30 minutes or more of boiling[26], and ATBC retention in quartered OSP varieties ranged from 70–81% after 20 minutes of boiling[21]. For other varieties including raw, whole unpeeled CNPH 1007, CNPH 1194, CNPH 1202, and CNPH 1205, 45–65 minutes of boiling generally resulted in BC retention of 75–103%[25]. Boiling whole Kamala Sundari, BARI SP 4, and BARI SP 5 with lemon juice to prevent oxidation also resulted in high retention (80–99%)[30]. Between whole unpeeled Tainung, SPK004, and Zapallo varieties boiled for 15–39 minutes, SPK004 retained the highest ATBC content of 128% (7830 µg/100 g FW), followed by 90% and 83% for Zapallo and Tainung (3570 µg/100 g and 9200 µg/100 g)[29]. Retention of over 100% may be due to leaching of solids and enhanced extraction of carotenoids upon heating[31].

Steaming is another commonly used preparation for OSP. Steaming peeled, cubed Kulfo or Tulla OSP resulted in the highest BC retention (94–96%) if steamed for 10 minutes, but BC true retention decreased for longer periods (as low as 54% at 50 minutes) [26]; similar results were shown for Yanshu No. 5 variety steamed from 10 to 50 minutes [28]. Retention values ranged between 87–102% after steaming whole, unpeeled CNPH 1007, CNPH 1194, CNPH 1202, and CNPH 1205 for 45–65 minutes in another study[25]. Steaming quartered OSP varieties Ejumula and several variants of SPK004 (see Table 4) in banana leaves for 30 min yielded ATBC retention values of 72–84% (59.3–249.9 µg/g DW), depending on variety. Steaming for 20 minutes and then oven-drying Yanshu No. 5 for 5 hours further decreased BC retention.

Deep-frying raw OSP, including Ejumula, SPK004, SPK004/6/6, SPK004/6, SPK004/1/1, SPK004/1, resulted in about 76–80% retention of ATBC (103.1–253 µg/g DW)[21]. Of these, the highest absolute ATBC content was retained in SPK004/6 (253.1 µg/g DW, compared to 103.1–210.3 µg/g DW across the other varieties).

Out of the drying methods, hot air, solar, and crossflow resulted in the highest retention values, suggesting these may be preferred. It appears that leaving OSP whole and unpeeled during boiling and steaming may be advantageous in retaining ATBC and BC. Alternatively, boiling or steaming smaller pieces of peeled potato for a shorter duration may also serve to preserve BC content. For frying and deep frying, fewer studies were done, but it appears variety is more

influential on retention values, which were lower than the highest retention found for boiling and steaming.

While OSP flour is not as commonly used in cooking, one study examined BC retention in fresh OSP milled into flour and blended with wheat. Milling oven-dried CNPH 1007, CNPH 1194, CNPH 1202, and CNPH 1205, into flour generally resulted in BC retention of 55–70%[25]. Extruding OSP flour maintained 47 to 99% of BC in the Beauregard Gold II (New) variety; retention values depended on moisture levels of 30, 35, or 40% and screw speeds ranging from 150-300 rpm[32]. Low BC retention (47% was observed at 30% moisture (screw speed unspecified), while the highest retention was found using 40% moisture, perhaps due to the fact that at lower extrusion moistures, melt temperature and viscosity are higher, leading to degradation of carotenoids and nutrients[32].

Some studies examined the retention of BC or ATBC in OSP products used as ingredients in other foods such as chapati, mandazi, cakes, and porridge. One study examined tunnel-dried, chipped Ejumula OSP which was milled and then made into chapati, mandazi, or porridge; true retention values ranged between 70–97%, 80-89%, and 69–93% respectively[33]. This suggests that fresh OSP flour retains ATBC adequately even after processing into a food product. In the Vita and Kabode varieties, retention of ATBC from OSP purees as an ingredient in chapati ranged from 81–82% (11.6–17.5 µg/g FW) in Vita and from 84-85% (36.2–37.9 µg/g FW) in Kabode [34]. Steaming Yanshu No.5 for 40 minutes and then frying OSP-based cakes in oil for 1 minute improved or maintained similar BC retention. This may be due to short-term frying-related changes in the cell wall structure that can allow increased beta-carotene release during the cooking process [28].

ATBC or BC: Processing and Storage. Boiling raw OSP and storing for 15 days resulted in slightly lower BC true retention values compared to raw OSP stored for 15 days at an unknown temperature (77–88%, 1.78–4.86 mg/100g), perhaps due to thermal and enzymatic degradation[20]. In another study, soaking, boiling, mashing, and storing OSP (8 variants of the DLP varietal) at -80 ºC resulted in BC retention values >100%[27].

Storing Beauregard OSP flour in various packaging and conditions—including polyester, metallic polyester, low density polyethylene, aluminium foil, and/or polyamide (nylon); with or without vacuum; in lit conditions (fluorescent lamps) at 23 ± 2 ºC and 70 ± 5% RH, or darkness at 25 ± 2 ºC and 75 ± 5% RH—for 50 to 360 days resulted in a wide range of BC retention values[35]. Low density polyethylene was included as a barrier to water vapor; aluminium foil and polyamide were included as barriers to oxygen. Briefly, the highest BC retention (>100%) value was observed at 360 days' storage, using polyester, aluminium foil, and polyethylene packaging with vacuum (light levels were not described) and was the only packaging to result in at least 100% retention of BC. Metallic polyester and polyethylene packaging with vacuum-sealing maintained retention values at over 90% by day 240, ultimately decreasing to 73% at day 360. Other packaging types and light levels (see **Supplementary Table 1** and online **Micronutrient Retention Dashboard**) resulted in a sharp decrease by day 50 which continued to decrease until day 360, reaching around 15% retention. This translates to a maximum ~39,590 µg BC/100 g, compared to levels as low as 5,000 µg/100 g after 360 days. Clearly, type of packaging is critical to maintain BC levels in OSP flour.

One study examined the storage of Ejumula OSP dried chips, stored for 88 days in ambient temperatures in a jar in the dark[36]. This resulted in about 41% ATBC retention (74.6 µg/g DW), which followed first-order beta carotene degradation kinetics.

In another study, a USA-based variety OSP was processed into dry flakes and then stored in various packaging for 30 to 210 days[37]. Shorter storage times resulted in greater BC apparent retention. Oxygen-permeable packaging and long duration of storage was detrimental to BC retention (33% retention or 13.2 µg/g DW), while using an oxygen-impermeable packaging type or an oxygen scavenger improved retention. The highest retention of 113% (45 µg/g DW) was found when OSP flakes were packaged with laminate film (oxygen barrier) made of saran-coated biaxially-oriented nylon and linear low density polyethylene sealant, with oxygen absorber sachet (Type/Size ZPT-100) in air headspace, stored in the dark at 23ºC and relative humidity 55-60%, for 120 days. It was unclear what caused retention values of over 100% in this study; authors surmised perhaps it was due to moisture loss during storage, but this was not supported by the study.

Cassava

BCE: Fresh storage. No studies examined BCE retention after storing cassava immediately after harvest, likely because cassava cannot last longer than 5 days without being processed (unless waxed).

BCE: Processing. Boiled cassava, from four cassava varieties, MVZ2011B/360, MVZ2012/044, Kindisa, or Vuvu, resulted in high apparent values (97.0–102.6%) and high true retention values, between 93–97% (3.76–24.22 µg/g DW), with MVZ2011B/360 yielding the highest absolute BCE content[38].

Eba, a cassava dish involving a two-day fermentation, toasting, milling, sieving, and cooking with hot water, was made using two varietals, SM 3765-15 and SM 3767-84. SM 3765-15 resulted in 75% retention of BCE, while SM 3767-84 resulted in 46%[39].

Porridge made with oven-dried cassava with a 2-day fermentation period improved BCE retention in SM 3765-15 and SM 3767-84 compared to omitting the fermentation step (30% vs 15% and 54% vs 43%, respectively)[39].

Chikwangue, a dish using grated cassava fermented for 3 days, smashed, sieved, pounded, partially boiled, kneaded, and steamed in leaves for 40 minutes, was made with four cassava varieties, Vuvu, Kindisa, MVZ2012/044, MVZ2011B/360, ~~or~~ resulted in low BCE apparent retention values, between 22–36%, and lower true retention values, between 4–18% (0.72–1.40 µg/g DW)[38]. MVZ2011B/360 yielded the highest absolute BCE content.

Fufu, a dish using chipped cassava fermented for 3 days, dried into cossettes (in this case, microcassettes), milled, and boiled, was made with four cassava varieties, Vuvu, Kindisa, MVZ2012/044, MVZ2011B/360, resulted in low BCE apparent retention values (1.5–5.6%) and true retention values, between 0.80–3.10% (0.10–0.45 µg/g DW)[38].

Please see **Supplementary Table 1** to review the true retention of ATBC in intermediate products made with cassava[38, 40].

BCE: Processing and Storage. No studies examined processing with post-processing storage of any biofortified cassava final food products. Please see **Supplementary Table 1** to review the true and apparent retention of BCE in intermediate products made with cassava and stored[38].

Pearl millet

Iron: Processing. Iron retention after soaking pearl millet for 12 or 24 hours in various grain:water ratios (1:2 or 1:5) was examined using varieties MAHYCO 204, ICTP 8203, H6M33, 8203 Nirmal-Mahabeej, and Mahalakshmi 504[41]. Contamination as indicated by aluminum concentration was not reported. The highest iron retention (87%, 4.1 mg/100 g) was found for soaking 8203 Nirmal-Mahabeej for 12 hours in a grain:water ratio of 1:5, closely followed by the same method for H6M33 (86%, 3.8 mg/100 g). The highest absolute iron content (4.6 mg/100 g) was found in ICTP 8203 using the same method. The lowest iron retention (52%, 3 mg/100 g) was observed in ICTP 8203 soaked for 24 hours in a grain:water ratio of 1:5, and in general soaking for 24 hours resulted in lower iron retention than 12 hours.

Adding a 48-hour germination step to the soaking method tended to lower the iron retention, for example from 87% to 66% for 8203 Nirmal-Mahabeej[41]. Malting soaked pearl millet for 6 minutes instead of germinating similarly lowered iron retention, from 87% to 63% for 8203 Nirmal-Mahabeej. Steeping and fermenting for 8 h and forced draught oven drying Dhanashaki or ICMH 1201 resulted in high iron retention (96 and 93%; 9.25 and 8.28 mg/100 g, respectively)[42]. Germinating raw AHB 1200 Fe flour for 72 hours followed by oven-drying at 50 ºC yielded 95% iron retention (7.8 mg/100g) in one study[43]. Neither contamination nor aluminum concentration was not reported in these studies.

Parboiled and forced-draught oven-dried Dhanashaki or ICMH 1201 resulted in high iron retention (103 and 92%; 9.80 and 8.15 mg/100 g, respectively)[42].

Decorticating (removing the millet husk) Dhanashakti, ICMH 1201, GB8735, and Tabi resulted in about 60–88% iron retention (3.9–7.2 mg/100 g DW) compared to raw whole pearl millet[42, 44]. However, in one of these studies, dishes prepared from the decorticated or whole pearl millet tended to have higher iron content than the raw grains, indicating iron contamination; the source of contamination may be less thorough washing of grains, iron content in water stored in metal tanks used for cooking, dust during sundrying of the grains, and rusty cooking utensils; however, aluminum concentration was not assessed.

Iron: Processing and Storage. Parboiling, forced-draught oven-drying, air cool milling into flour, and storing raw decorticated Dhanashaki or ICMH 1201 for ≤1 month (10 ºC) resulted in ≥100% iron retention for ICMH 1201 (5.34 µg/100 g) and 88% iron retention for Dhanashakti (6.31 mg/100g)[42]. Repeating the same steps except replacing parboiling with a steeping and fermentation step had varying effects on iron retention: for ICMH 1201, retention decreased slightly to 98% (5.18 mg/100 g), while for Dhanashakti, retention increased to 93% (6.7 mg/100 g). As decortication results in 60–88% of iron retention, additional processing steps may further reduce iron retention; however, the effect of additional steps appears to depend upon variety. Again, aluminum concentration was not reported.

Post-processing storage for ≤1 month did not negatively impact iron retention in pearl millet.

Zinc: Processing. Zinc retention after soaking pearl millet for 12 or 24 hours in various grain:water ratios (1:2 or 1:5) was examined using varieties MAHYCO 204, ICTP 8203, H6M33, 8203 Nirmal-Mahabeej, and Mahalakshmi 504[41]. The highest iron retention (94%, 3 mg/100 g) was found for soaking Mahalakshmi 504 for 12 hours in a grain:water ratio of 1:5; this decreased to 91% upon changing the grain:water ratio to 1:2. The highest absolute zinc content

(4 mg/100 g) was found in 8203 Nirmal-Mahabeej using the same method with grain:water 1:2. The lowest zinc retention (48%, 2.9 mg/100 g) was observed in ICTP 8203 soaked for 24 hours in a grain:water ratio of 1:2 or 1:5. In general, soaking for 24 hours resulted in lower zinc retention than 12 hours. Adding a 48-hour germination step to the soaking method tended to lower the zinc retention, for example from 94% to 75% for Mahalakshmi 504. Germinating raw AHB 1200 Fe flour for 72 hours followed by oven-drying at 50 ºC yielded 93% zinc retention (3.72 mg/100g) in one study[43] .Malting soaked pearl millet for 6 minutes instead of germinating similarly lowered zinc retention, from 94% to 69% for Mahalakshmi 504. Steeping and fermenting for 8 h and forced draught oven drying Dhanashaki or ICMH 1201 resulted in high zinc retention (100 and 97%; 4.81 and 4/22 mg/100 g, respectively)[42].

Parboiled and forced-draught oven-dried Dhanashaki or ICMH 1201 resulted in high zinc retention (105 and 97% 5.06 and 4.21 mg/100 g, respectively)[42].

Decorticating Dhanashakti and ICMH 1201, resulted in about 95–103% zinc retention (4.12–4.97 mg/100 g DW) compared to raw whole pearl millet[42, 44].

Zinc: Processing and Storage. Parboiling, forced-draught oven-drying, air cool milling into flour, and storing raw decorticated Dhanashaki or ICMH 1201 for ≤1 month (10 ºC) resulted in 96% zinc retention for ICMH 1201 (4.76 µg/100 g) and 98% zinc retention for Dhanashakti (4.02 mg/100g)[42]. Repeating the same steps except replacing parboiling with a steeping and fermentation step had varying effects on iron retention: for ICMH 1201, retention increased to 103% (4.26 mg/100 g), while for Dhanashakti, retention increased to 98% (4.88 mg/100 g). Decortication results in 95-103% of zinc retention, and the effect of additional steps appears to improve zinc retention.

Post-processing storage for ≤1 month did not negatively impact zinc retention in pearl millet.

Beans

Iron: Processing. Soaking a combination of biofortified beans—BIO 101, BIO 107, and ICTA Chortí beans for 18 hours resulted in 98% iron retention (86.51 µg/g). Soaking and then boiling improved iron retention (87% vs 82%, 76.61 vs 72.63 µg/g DW)[45]. Iron true retention was similar whether or not a soaking step was included prior to boiling and then refrying (93% vs 91%, 93.4 vs 91.2 µg/g DW) [45]. Why boiling and then refrying resulted in higher iron retention than boiling alone may be due to that cooking broth was used during refrying, which added additional iron to the beans; another explanation may be that leakage of solubles into the water increased the retention values[45]. Given the overall high iron retention after boiling or refrying, soaking may be ideal for boiling but is not as crucial for refrying.

Varieties including Supremo, Porto Real, Pirata, and Brazil were processed into flour by soaking in water which was then discarded, autoclaving, lyophilizing, and grinding[46]. All four varieties achieved iron retention ≥99% (71.69–88.48 mg/kg). Beans were grown under strictly controlled conditions, and it is unclear as to what contributed to the high iron retention observed given that soaking water was discarded, which itself may contain leached iron[47].

Raw flour of bean varieties ROBA1 and K131 was either malted/roasted or extruded[48]. Malting/roasting raw flour yielded 83 and 71% iron retention, respectively, while extrusion resulted in ≥100% iron retention. Such processes are reported to reduce the level of antinutrients such as phytates and phenols, and improve cooking characteristics and

physiochemical properties of beans[49, 50]. Extrusion, particularly, reduces paste viscosity and enhances nutrient content of dishes from beans [51].

Iron: Processing and Storage. No studies examined iron retention after processing and then storing beans.

Zinc: Processing. Zinc was retained at 93% after soaking a combination of biofortified beans—BIO 101, BIO 107, and ICTA Chortí—for 18 hours. Zinc retention after boiling these beans was slightly lower if a soaking step was included prior to boiling (78% vs 81%, 30.46 vs 31.71 µg/g DW)[45]. Similarly, zinc retention was lower if a soaking step was included prior to boiling and then refrying (86% vs 91%, 33.47 vs 35.70 µg/g DW)[45]. Soaking may be helpful for iron retention, but appears to negatively impact zinc retention.

Varieties including Supremo, Porto Real, Pirata, Brazil were processed into flour by soaking, autoclaving, lyophilizing, and grinding[46]. Supremo, Porto Real, and Brazil achieved zinc retention between 92–105% (32.75–34.76 mg/kg) while Pirata showed 75% zinc retention. Again, it was unclear as to what contributed to retention values of >100%.

Raw flour of bean varieties ROBA1 and K131 was either malted/roasted or extruded[48]. Malting/roasting raw flour yielded 86 and 100% zinc retention, respectively, while extrusion resulted in 85–92% zinc retention.

Zinc: Processing and Storage

No studies examined zinc retention after processing and then storing beans.

Rice

Iron: Processing. Paddy rice is the stage after threshing (as opposed to panicles, the stage before threshing) but before any further processing[52]; the rice husk is still intact[53]. Drying several varieties (AM1, AM143, AM180, Am65, AM72, ARB6, Jeerigesanna, Sebati, Vandhana) of unsoaked, sun-dried paddy rice (10–12% moisture) (11.00–13.77 mg/kg) to 10% moisture and de-husking into non-polished brown rice retained about 57–85% of iron (7.70–11.87 mg/kg), with Jeerigesanna yielding the highest absolute iron amount, yet lower iron retention value of 73%[53]. Polishing paddy rice to 5% degrees of milling resulted in 30–51% retention of iron (4.78–6.07 mg/kg), with AM180 yielding the highest iron content. Further polishing paddy rice to 10% degrees of milling resulted in 27–41% iron retention (4.70–5.00 mg /kg), or about a 0.2–11% lower iron retained between 5% and 10% polishing depending on varietal; at this step, most varietals had the same iron content.

In another study, iron retention of biofortified three rice varietals grown in two different locations, Palmira and Santa Rosa, in Colombia was examined[52]. This study examined iron retention after soaking and dehulling paddy rice, with or without involving a parboiling step. Soaking and dehulling paddy into brown rice resulted in 100% iron retention for varieties BRRI dhan64 (BF1), BF14AR035 (BF2), BF14AR050 (BF3) (10.50–12.30 µg/g) across both locations. Polishing soaked, dehulled paddy rice to 7.5% degrees of milling resulted in much lower iron retention values of 32–47% (3.60–5.70 µg/g)[52]. BF3 yielded the highest absolute iron content in both Palmira and Santa Rosa (5.20 and 5.70 µg/g, respectively). Additional polishing to 10% yielded retention values of 21–33% (2.30–3.70 µg/g) compared to unsoaked paddy rice[52]. In Palmira,

BF3 yielded the highest absolute iron content (3.70 µg/g) while in Santa Rosa, BF1 yielded the highest amount (also 3.70 µg/g).

Parboiling paddy rice for 13 minutes followed by gradual cooling and dehulling into brown rice resulted in iron retention values around 91–100% (10.30–12.50 µg/g) across both locations. Polishing this parboiled rice to 7.5% degrees of milling resulted in similar values as non-parboiled rice, between 31–39% iron retention (3.50–4.40 µg/g)[52]. Again, in Palmira, BF3 yielded the highest absolute iron content (4.50 µg/g) while in Santa Rosa, BF1 yielded the highest amount (4.4 µg/g). Polishing parboiled to 10% degrees of milling resulted in lower iron retention of about 16–30%. BF1 yielded the highest absolute iron content across both locations.

Parboiling for 16 minutes followed by immediate cooling resulted in similar retention values as 13 minutes' parboiling, between 91–104% (10.20–12.60 µg/g). Adding a 7.5% degrees of milling polish step similarly decreased iron retention values between 29–36%, with BF3 yielding the highest iron content in both locations (4.60 and 3.70 µg/g in Palmira and Santa Rosa, respectively).

Iron: Processing and Storage. No studies examined iron retention after processing and then storing rice.

Zinc: Processing. Several rice varieties (AM1, AM143, AM180, Am65, AM72, ARB6, Jeerigesanna, Sebati, Vandhana) of unsoaked, sun-dried paddy (10–12% moisture) (11.00–13.77 mg/kg) to 10% moisture and de-husking into non-polished brown rice retained about 81–100% of zinc (22.09–33.92 mg/kg), with AM65 yielding the highest absolute zinc amount, yet lower zinc retention value of 75%[53]. Polishing paddy rice to 5% degrees of milling resulted in 46–82% retention of zinc (12.49–24.70 mg/kg), with AM65 yielding the highest zinc content despite having a lower retention value of 55%. Further polishing paddy rice to 10% degrees of milling resulted in 44–80% zinc retention (11.95–24.44 mg /kg), or about 2% lower zinc retained between 5% and 10% polishing depending on varietal.

Taleon and colleagues examined zinc retention of biofortified three rice varieties grown in either Palmira and Santa Rosa, in Colombia[52]. First, this study examined zinc retention after soaking and dehulling paddy rice, with or without parboiling. Soaking and dehulling paddy into brown rice resulted in ~100% zinc retention for varieties BRRI dhan64 (BF1), BF14AR035 (BF2), BF14AR050 (BF3) (16.88–35.10 µg/g) across both locations. Polishing soaked, dehulled paddy rice to 7.5% degrees of milling resulted in lower zinc retention values of 78–85% (14.20–33.80 µg/g)[52]. BF3 yielded the highest absolute zinc content in both Palmira and Santa Rosa (17.50 and 33.80 µg/g, respectively). Further polishing to 10% yielded retention values of 75–89% (12.10–31.20 µg/g) compared to unsoaked paddy rice[52]. In Palmira, BF2 yielded the highest absolute zinc content (15.90 µg/g) while in Santa Rosa, BF3 yielded the highest amount (31.20 µg/g).

Parboiling paddy rice for 13 minutes, gradual cooling and dehulling into brown rice resulted in zinc retention of around 98–107% (18.20–34.40 µg/g) across both locations. Polishing this parboiled rice to 7.5% degrees of milling resulted in slightly lower values compared to non-parboiled rice, between 62–77% zinc retention (11.60–23.20 µg/g)[52]. BF2 yielded the highest absolute zinc content in Palmira and Santa Rosa (15.40 and 23.20 µg/g, respectively). Polishing parboiled to 10% degrees of milling resulted in lower zinc retention of about 54–63% (10.4–20.80 µg/g), with rice grown in Santa Rosa having higher zinc content (19.00–20.8 µg/g) than rice grown in Palmira (10.40–13.10 µg/g).

Parboiling for 16 minutes and mmediate cooling resulted in similar retention values as 13 minutes' parboiling, between 101-108% (17.20–36.80 μg/g). Adding a 7.5% degrees of milling polish step similarly decreased zinc retention values between 63–77%, with rice grown in Santa Rosa yielding the highest zinc content (20.30–24.90 μg/g) compared to Palmira (11.50–15.50 μg/g).

Zinc: Processing and Storage. No studies examined zinc retention after processing and then storing rice.

Wheat

Zinc: Processing. One study examined zinc-biofortified wheat[54] (**Supplementary Table 1)**; specifically, a combination of Durango (DGO95.1.17), Durango (DGO95.3.2), Chihuahua (CHIH95.2.1), Chihuahua (CHIH95.2.47), Chihuahua (CHIH95.3.47), Jalisco (JAL95.4.10), LGP2, LGP12 was milled into flour using either 95% or 80% extraction. Milling using 95% extraction resulted in 98% retention of zinc, while milling using 80% extraction resulted in 58% retention of zinc.

Zinc: Processing and Storage. No studies examined zinc retention after processing and then storing wheat.

Supplementary Tables

Supplementary Table 2. Characteristics of included studies: maize							
**Varietal**	**Micro-nutrient**	**Micronutrient analysis method**	**Starting product**	**Processing**	**Ending product**	**Country**	**Ref**
PVAH-62	TCC	"Standard methods"	Raw kernels	Mashed, fermented 1 day, dewatered, sieved, roasted	Amahewu	South Africa	[55]
Oba-Super-II	BCX	HPLC	Raw cob, DAP 20	Roasted ± husk	Roasted cob	NR	[56]
			Raw cob, DAP 27				
			Raw cob, DAP 34				
BRS4104	BC<br>TCC<br>PVA	HPLC	Raw cob, milky stage	Microwaved	Microwaved cob	Brazil	[8]
				Pressure cooked with water	Pressure cooked cob		
				Boiled in pot with lid with water	Boiled		
				Boiled in pot without lid with water			
				Dehusk, sanitize, store in polystyrene trays covered with PVC film @5ºC for 9 days	Stored cob		
				Dehusk, sanitize, store in vacuum-packed ears in multilayered polynylon @5ºC for 9 days			
PVAH 79-100<br>PVAH 1-26<br>PVAH 27-49<br>PVAH 50-75	PVA/BCE	UPLC	Super maize meal	Roller-milled, boiled	Porridge	South Africa	[13]
NR ("PVA enriched maize, 10 genotype combo)	PVA	UPLC	Raw cob	Boiled	Boiled cob	Mexico	[9]

NR ("high kernel zinc maize, 10 genotype combo")	Zn			Boiled	Boiled cob		
				Dried	Dried cob		
‘23–6 × 2–9’	TCC<br>BC	HPLC	Raw dehusked cob	Stored 3 months @ -80 ºC	Stored	Australia	[57]
'2–9 × 11–7'	TCC		Raw dehusked cob	Stored 5–15 days @ 4 ºC, then 3 months @ -20 ºC			
Pusa-PV-16-3<br>Pusa-PV-16-2<br>Pusa-PV-16-4	BC	HPLC	Raw flour	Ball-milled, boiled	Porridge	India	[58]
Pool 8A	BC<br>pVAC<br>TCC	HPLC	Raw kernels	Freezer-milled (0.5 mm), stored in aluminum pouch for 10–180 days @4ºC or 37ºC	Flour	Rwanda	[16]
				Freezer-milled (0.5 mm), stored in laminated paper bag for 10–180 days @4ºC or 37ºC			
				Freezer-milled (0.5 mm), stored in double-layered polyethylene bag for 10–180 days @4ºC or 37ºC			
				Rotor-milled (0.5 mm), stored in aluminum pouch for 10–180 days @4ºC or 37ºC			
				Rotor-milled (0.5 mm), stored in laminated paper bag for 10–180 days @4ºC or 37ºC			
				Rotor-milled (0.5 mm), stored in double-layered			

				polyethylene bag for 10–180 days @4ºC or 37ºC			
NR (“orange maize”)	BC	Modified HPLC	Flour (whole grain)	Milled	Flour (refined)	Zambia	[59]
(DeExp x Cl.7) x BC Orange hybrid	BC	HPLC	Flour	Fermented	Flour	USA	[60]
				Fermented, cold-boiled	Porridge		
				Cold-boiled			
	BC ATBC		Dried kernels	Soaked, dehulled, wet-milled, fermented	Flour		
				Soaked, dehulled, wet-milled, fermented, cold-boiled	Porridge		
				Soaked, dehulled, wet-milled, cold boiled			
GV662A (HP1002) GV665A (HP1005)	BCE	HPLC	Raw kernels, dry (harvested at 7 months, 12% moisture)	Stored 15–180 days, ambient conditions	Stored raw kernels	Zambia	[4]
all (HP1001/2/3/5) HP1001 GV662A (HP1002) HP1003 GV665A (HP1005)			Raw kernels, green (harvested at 5 months, 61% moisture)	Dried	Dried kernels		
			Raw kernels, dry (harvested at 7 months, 12% moisture)	Boiled	Boiled kernels		
				Roasted	Roasted kernels		
				Hammer-milled	Mealie meal		
				Hammer-milled, boiled, simmered	Nshima		
				Hammer-milled, boiled	Porridge		
				Hammer-milled, cold water steeped, decorticated	Samp (raw)		

				Hammer-milled, cold water steeped, decorticated, washed, boiled	Samp (cooked)		
				Hammer-milled, boiled, simmered, stored 90 days	Nshima, stored		
				Hammer-milled, cold water steeped, decorticated, washed, boiled	Samp (cooked), stored		
				Hammer-milled, cold waterer steeped, decorticated, washed, boiled, stored 90 days	Porridge, stored		
OPVI<br>OPVII	ATBC<br>BCE<br>pVAC<br>TCC	LC	Raw, cob	Dried to 8.5% moisture, stored 2 months, @29ºC, humidity 30% PICS-OXY packaging	Stored cob	USA	[14]
				Dried to 8.5% moisture, stored 2 months, @29ºC, humidity 30% PICS-NOXY packaging			
				Dried to 8.5% moisture, stored 2 months, @29ºC, humidity 30% woven packaging			
C17 × DE3<br>Hi27 × CML328<br>2013 Orange ISO<br>2015 Orange ISO<br>[KUI carotenoid syn-FS17-3-1-B-B-B-B-B-B-B-B] × [(MAS[206/312]-23-2-1-1-B-B-	ATBC<br>TCC<br>PVA/BCE<br>ATBC		Flour	Steeped, fermented 24h, 72h, or 120h at 27ºC, milled, filtered, freeze-dried 3 days, milled to fine meal	Flour (refined, fermented)	USA	[12]
				Steeped, milled, fermented 24h, 72h, or 120h @ 27ºC, filtered, freeze-dried	Ogi		

B/[BETASYN]BC1-6-5-1xFloridaASYN#-B)-B-1-3-B-B-B]				3 days, milled to fine meal, boiled, incubated, stored @-80ºC			
2012 Orange ISO Selected A<br>(CI7×DE3)×2010-Orange-Isolation<br>CI7×DE3<br>Hi27×CML328<br>[KUI carotenoid syn-FS17-3-1-B-B-B-B-B-B-B-B] × [[(CML297-B × CML 324-B) × (KUI3 × SC55)] S1]<br>[KUI carotenoid syn-FS17-3-1-B-B-B-B-B-B-B-B] × [(MAS[206/3	ATBC<br>pVAC<br>TCC	HPLC	Raw kernels	Vacuum oven-dried (40ºC for 48h), stored 53 weeks, @4ºC, 64% RH, NaCl solution	Dried kernels, treated, stored	USA	17
				Vacuum oven-dried (40ºC for 48h), stored 53 weeks, @22.5ºC, 59% RH, NaCl solution			
				Vacuum oven-dried (40ºC for 48h), stored 27 weeks, @55ºC, 0.65% RH, NaCl solution			
C17 × DE3<br>Orange ISO	ATBC<br>TCC<br>PVA		Raw kernels	Conditioned, roller-milled, degerminated	Flour (refined)		
				Storage 15–60 days @22.5ºC, 11% RH	Flour		
					Grits		
					Meal		
Orange ISO	ATBC<br>TCC	HPLC	Flour (refined)	Extrusion cooking (150, 225, or 300 rpm), Moisture 25%, 30%, or 35%	Flour (refined), extruded	USA	61
10 MAK 7-5<br>10 MAK 7-7<br>10 MAK 7-8	BC<br>PVA<br>TCC	HPLC	Raw kernels	Cleaned, degerminated, roller-milled	Mealie meal (raw)	South Africa	11
				Cleaned, degerminated	Samp (Raw)		

				Cleaned, degerminated, roller-milled, simmered	Phutu		
				Cleaned, degerminated, roller-milled, simmered, meal paste	Porridge		
				Cleaned, degerminated, steeped, boiled	Samp (cooked)		
CYH1<br>CYH2<br>BEH1<br>BEH2<br>BEH3<br>BEH4<br>BEH5<br>BEH6<br>BEH7	BCE	UPLC	Raw kernels	Traditional nixtamalization	Flour	Mexico	[10]
					Tortilla dough		
					Tortilla		
				Extrusion nixtamalization	Flour		
					Tortilla dough		
					Tortilla		
UMI 1200 B	BC	HPLC	Flour	Rinsed, drained, heated 2 min in popcorn maker, cyclone grinder-milled	Popcorn	India	[58]
				Dry-milled, ingredients, steamed, kneaded, extruded, steamed, cooled, oven-dried @45ºC for 3h	Chapati		
				Dry-milled, ingredients, roasted	Roti		
				Dry-milled, ingredients, roasted	Noodles		
F2 seed of C17/Deexp KUI synthetic	BC	HPLC	Raw cob	Dried, hammer-milled, sieved, stored 2–12 months @ -20–37 ºC	Flour	USA	[62]
F2 seed of C17/Deexp	AEBC<br>TCC	HPLC	Flour (whole grain)	Boiled, simmered	Nshima		

			Flour (refined grain)				
			Tortilla dough (whole grain)	Fried (150ºC for 1.5 min)	Tortilla		
			Tortilla dough (refined grain)				
			Puffs (whole grain, uncooked)<br>Puffs (refined grain, uncooked)	Deepfried	Puffs		
			Muffins (whole grain, uncooked)<br>Muffins (refined grain, uncooked)	Baked	Muffins		
GV664A	BCE	HPLC	Raw cob	Dried 3 days (shade, ambient conditions), shelled, stored 25–180 days in aluminum bags	Stored	Zambia	[15]
				Dried 3 days (shade, ambient conditions), shelled, stored 25–60 days in PICS bags			
				Dried 3 days (shade, ambient conditions), shelled, stored 30–180 days in metal silo, candle sealed bags			
				Dried 3 days (shade, ambient conditions), shelled, stored 60–180 days in metal silo (no candle seal)			

				Dried 3 days (shade, ambient conditions), shelled, stored 5–180 days in woven bags			
				Dried 3 days (shade, ambient conditions), shelled, stored 10-120 days in single layer polyethylene bags	Hammer meal		
				Dried 3 days (shade, ambient conditions), shelled, stored 10–120 days, single layer polyethylene bags	Breakfast meal		
				Dried 3 days (shade, ambient conditions), shelled, stored 30–120 days in PICS bags	Hammer meal		
				Dried 3 days (shade, ambient conditions), shelled, stored 30–120 days in multilayer polyethylene bags	Breakfast meal		
				Dried 3 days (shade, ambient conditions), shelled, stored 10–120 days in aluminum bags	Hammer meal		
				Dried 3 days (shade, ambient conditions), shelled, stored 10–120 days in aluminum bags	Breakfast meal		
AEBC, all-E-beta carotene; ATBC, all-trans beta-carotene; BC, beta-carotene; BCE, BC equivalents; BCX, beta-cryptoxanthin; DAP, days after pollination; h, hours; HPLC, high-performance liquid chromatography; LC, liquid chromatography; min, minutes; NR, not reported; PICS, Purdue							

Improved T Crop Storage; PICS-NOXY, Purdue Improved T Crop Storage (PICS) bags; PICS-OXY, Purdue Improved T Crop Storage (PICS) bags, oxygen scavenger; PVA, provitamin A; pVAC, provitamin A content; RH, relative humidity; rpm, rotations per minute; TCC, total carotenoid content; UPLC, ultra Performance Liquid Chromatography; Zn, zinc<br>*Country refers to where crops were grown

Supplementary Table 3. Characteristics of included studies: orange sweet potato

Varietal	Micro-nutrient	Micronutrient analysis method	Starting product	Processing	Ending product	Country*	Ref
Beauregard	TCC<br>BC	HPLC	Flour	Stored 50-360 days in various packaging, light/dark settings	Stored flour	Brazil	[35]
Rubina®, Agrexco	ATBC<br>TCC	HPLC	Raw chips	Cross flow-dried (2h), fan operated greenhouse solar-dried (8h), or sun-dried (8h)	Chips	USA	[63]
Ejumula<br>Kakamega	TCC	HPLC	Raw chips	Dry or wet weather; tent-dried, tunnel-dried, or sun-dried	Chips	Uganda, Mozambique	[63] [3]
				Stored 125 days, various packaging (black or clear PE bag with or without simple knot)	Stored chips		
MGCL	TCC	HPLC	Raw chips	Dry weather; sun-dried	Chips	Uganda, Mozambique	[3]
Resisto	TCC	HPLC	Raw chips	Dry weather; sun-dried or tunnel-dried	Chips	Mozambique	[64]
Ejumula<br>Kakamega	TCC	RP-HPLC	Raw	Stored 125 days in various packaging	Stored raw	Uganda, Mozambique	[22]
MCGL01	TCC		Raw	Rotary disk chipper thin or thick, or handsliced, dried in dry or wet weather	Chips		
Ejumula	TCC		Raw	Stored 4 or 6 months; pretreated with either 0.5% $Na_2S_2O_5$, 0.5% citric acid, 1% salt, or water	Pretreated and stored		
OSP (USA variety)	TCC<br>ATBC	RP-HPLC<br>UV-VIS	Raw chips	Hot air-dried (2h), solar-dried (8h), or sun-dried (8h)	Dried	USA	[3, 22] [63] [3]
Rubina®, Agrexco	ATBC		Raw chips	Chipped, cross-flow dried	Chips		
				Crimped, cross-flow dried			
				Chipped, sun-dried			

				Chipped, greenhouse-dried			
MGCL01	TCC	HPLC	Raw chips	Cut thinly, thickly, or in slices; tunnel-dried, open-dried, or shade-dried	Chips	Mozambique	[64]
Ejumula	ATBC	HPLC	Flour	Chipped, tunnel-dried, milled, wheat:OSP 70:30, oil, roasted	Chapati	Uganda	[33]
				Chipped, tunnel-dried, milled, wheat:OSP 70:30, deep-fried	Mandazi		
				Chipped, tunnel-dried, milled, maize:soybean:OSP 30:35:35, boiled	Porridge		
Ejumula	ATBC<br>TCC	RP-HPLC	Dried chips	Stored 88 days at ambient temps in a jar in the dark in the UK or Uganda, or stored 125 days in LPDE bags in Uganda	Stored chips	Uganda	[36]
Ejumula<br>SPK004<br>SPK004/6/6<br>SPK004/6<br>SPK004/1/1<br>SPK004/1	TCC	UV-VIS	Raw	Dried-open air sun (28.3 ± 5.6 ºC; 55.1 ± 23.8% RH)	Dried chips	Uganda	[23]
			Dried chips	Dried-ambient temps, storage 125 days,black PE bags	Stored dried chips		
Ejumula<br>Kakamega	TCC	UV-VIS	Raw	Chipped-rotary disk (thin slices), quartered, soaked in: 1% salt solution (pH=6.7) *or* 1% ascorbic acid (pH=2.5) *or* 1% sodium metabisulfite (pH=4.5) *or* blanched (internal 60-82ºC); sun-dried until presence of lour and cracking noise when crushed	Chips, pretreated<br>Chips, blanched	Uganda	[65]
Ejumula	BC	HPLC	Flour	[Chipped, tunnel-dried, milled], wheat:OSP 70:30, oil, roasted	Chapati	Uganda	[33]

				[Chipped, tunnel-dried, milled], wheat:OSP 70:30, deep-fried	Mandazi		
				[Chipped, tunnel-dried, milled], wheat:OSP 70:30, boiled	Porridge		
Ejumula<br>SPK004<br>SPK004/6/6<br>SPK004/6<br>SPK004/1/1<br>SPK004/1	ATBC	HPLC	Raw, unpeeled	Peeled, boiled	Boiled	Uganda	[21]
				Peeled, steamed in banana leaves	Steamed		
				Peeled, deepfried	Deep-fried		
				Peeled, oven-dried	Dried		
				Peeled, solar-dried			
				Peeled, open air sun-dried			
CNPH 1007<br>CNPH 1194<br>CNPH 1202<br>CNPH 1205	BC	HPLC	Raw	Boiled	Boiled	Brazil	[25]
				Roasted	Roasted		
				Steamed	Steamed		
				Peeled, oven-dried, knife-milled	Flour		
Beauregard	TCC	UV-VIS	Chips	Stored 31 to 207 days in various packaging, with or without nitrogen, with or without oxygen scavenger	Stored chips	Brazil	[66]
S-61<br>S-594<br>S-1156<br>S-1281<br>SV-98<br>362-7<br>IGSP-15<br>CIPSWA-2<br>187017-1<br>440038<br>440127<br>420027<br>ST-14<br>Kamala Sundari<br>90/101	BC	AOAC method (NR)	Raw, peeled, shredded, stored 15 days	Boiled, stored 15 days	Boiled	India	[20]

S-61<br>S-594<br>S-1156<br>S-1281<br>SV-98<br>362-7<br>IGSP-15<br>CIPSWA-2<br>187017-1<br>440038<br>440127<br>420027<br>ST-14<br>Kamala Sundari<br>90/101	BC		Raw, peeled, shredded	Stored 15 days	Raw, stored		
Bophelo	Vitamin A	HPLC	Raw, grated	Oven-dried @70–80 ºC for 7 days, milled	Flour	South Africa	67
Kulfo<br>Tulla	BC	HPLC	Raw peeled	Steamed 10-50 min	Steamed	Ethiopia	26
				Boiled 10–50 min	Boiled		
KS-7<br>ST-14-1<br>ST-14-16<br>ST-14-34<br>ST-14-49<br>ST-14-53<br>ST-14-6<br>ST-14-9<br>ST-3-17<br>ST-3-22	TCC<br>BCC	Column chromate-graphy	Raw, unpeeled	Boiled	Boiled	India	24
				Peeled, thinly sliced, blanched, Oven-dried, Fried	Chips		
				Oven-dried (50-60ºC for 24-48 h)	Dried		
				Sun-dried (48h)	Dried		
DLP 194568.1<br>DLP 194512.7<br>DLP 192033.5<br>DLP 194583.2<br>DLP 189531.2<br>DLP 102025.3<br>DLP 189123.5<br>DLP 190094.2	BC	RP-HPLC	Raw, unpeeled	Soaked, boiled, mashed stored at -80ºC	Boiled	Peru	27
Dark OSP	ATBC	HPLC	Raw, unpeeled	Peeled, cubed (10mm x 10mm), fried in soybean oil for 1 min at 160ºC	Fried	Kenya	29

				Peeled, cubed (10mm x 10mm), fried in soybean oil for 3 min at 160ºC			
				Peeled, shredded (6 mm), fried in soybean oil for 1 or 3 min at 160ºC			
Kamala Sundari<br>BARI SP 4<br>BARI SP 5	ATBC	Spectro-photometric	Boiled	Unpeeled, boiled with lemon juice	Boiled	Bangladesh	[30]
Tainung<br>SPK004<br>Zapallo	ATBC	RP-HPLC	Raw, unpeeled	Unpeeled, boiled with lid until core temp 86 ºC (15-39 min), cooled, quartered, peeled, packed in Al foil pouches (PETP12/ALU9/LLDPE75, Danisco Flexible, Horsens, Denmark), stored -24ºC until analysis (time NR)	Boiled	NR	[68]
				Unpeeled, roasted (spherical grill with charcoal) for 30-90 min with regular rotation, until core temp of 86 ºC, cooled, quartered, peeled, packed in Al foil pouches (PETP12/ALU9/LLDPE75, Danisco Flexible, Horsens, Denmark), stored -24ºC until analysis (time NR)			
				Grated (0.2-0.3 cm chips), dried-shade (5h) until 6% moisture content, stored at 0 ºC for 2 days, stored at -24 ºC until analysis (time NR)			
Yanshu No. 5	BC	HPLC	Raw, peeled	Boiled 10, 20, 30, 40, or 50 min	Boiled	China	[28]

				Steamed 10, 20, 30, 40, 50 min	Steamed		
				Microwaved 10, 15, 20 min	Microwaved		
				Steamed 20 min, dried at 50ºC for 5 or 11 h	Steamed and dried		
				Steamed 40 min, shaped into cakes, fried in rapeseed oil for 1 min	Steamed cakes		
Kakamega 4 (SPK 004)<br>Japon Tresimesino Selecto (CIP420009)	TCC	HPLC	Raw	Boiled 30, 45, or 60 min	Boiled	Kenya	[31]
Camote Amarillo (CIP400014)<br>Japon Tremesino Selecto (CIP420009)<br>Teoboza (CIP420010)<br>Zapallo (CIP420027)<br>Mamala (CIP420004)<br>SPK004	TCC	Spectro--photometry	Raw, peeled	Chipped (2-4 mm thick), dried-forced air oven @65ºC to moisture content of 6-8%	Chips, dried	Kenya	[69]
				Chipped (2-4 mm thick), dried-forced air oven @65ºC to moisture content of 6-8%, stored 3, 6, or 11 months in a paper bag in ambient conditions	Chips, dried, stored		
Zapallo (CIP420027)	TCC		Raw, peeled	Boiled	Boiled		
				Chipped (2-4 mm thick), dried-forced air oven @65ºC to moisture content of 6-8%, stored 11 months in a paper bag in ambient conditions	Flour		
				Grated, mixed with (wheat flour, dry yeast, water, sugar, oil), baked 25-30 min after rising	Buns		

				Grated, mashed, mixed with (wheat flour (3:7 OSP:Wheat ratio), salt, baking powder, corn oil, water), rolled flat, grilled 30 s per side	Chapati		
				Grated, mashed, mixed with (wheat flour (3:7 OSP:Wheat ratio), sugar, baking powder, corn oil, water), kneaded, rolled, cut, deep-fried	Mandazi		
			Flour	OSP flour mixed with (wheat flour, dry yeast, water, sugar, oil), baked 25-30 min after rising	Buns		
				mixed with (wheat flour (3:7 OSP:Wheat ratio), salt, baking powder, corn oil, water), rolled flat, grilled 30 s per side	Chapati		
				mixed with (wheat flour (3:7 OSP:Wheat ratio), sugar, baking powder, corn oil, water), kneaded, rolled, cut, deep-frieds	Mandazi		
			Boiled 30 min	mixed with (wheat flour, dry yeast, water, sugar, oil), baked 25-30 min after rising	Buns		
				mixed with (wheat flour (3:7 OSP:Wheat ratio), salt, baking powder, corn oil, water), rolled flat, grilled 30 s per side	Chapati		
				mixed with (wheat flour (3:7 OSP:Wheat ratio), sugar, baking powder, corn oil, water), kneaded, rolled, cut, deep-fried	Mandazi		

OSP (USA varietal)	BC	RP-LC	Flakes stored in PE film after 1 day	Dried-drum, processed into dry flakes, packaged with laminate fim (O2 permeable) made of biaxially-oriented PP and LLDPE sealant, stored in the dark at 23ºC and RH 55-60%, storage 30 days, 60, 120, 210 days	Flakes, stored	Guatemala	[37]
				Dried-drum, processed into dry flakes, packaged with laminate film (oxygen barrier) made of saran-coated biaxially-oriented nylon and LLDPE sealant, stored in the dark at 23ºC and RH 55-60%, storage 30, 60, 120, 210 days			
				Dried-drum, processed into dry flakes, packaged with laminate film (oxygen barrier) made of saran-coated biaxially-oriented nylon and LLDPE sealant, under vacuum, stored in the dark at 23ºC and RH 55-60%, storage 120 or 210 days			
				Dried-drum, processed into dry flakes, packaged with laminate film (oxygen barrier) made of saran-coated biaxially-oriented nylon and LLDPE sealant, with oxygen absorber sachet (Type/Size ZPT-100) in air headspace, stored in the dark at 23ºC			

				and RH 55-60%, storage 60, 120, 210 days			
Vita Kabode	ATBC	HPLC	Puree	[Boiled unpeeled 1 hr @100ºC, cooled, pureed by hammer-mill, vacuum packed, stored at -20ºC], mixed with maize flour 50:50, mixed with water, cooked in hot water for 5 min, cooled, stored at -80ºC	Porridge	Kenya	[34]
				[Boiled unpeeled 1 hr @100ºC, cooled, pureed by hammer-mill, vacuum packed, stored at -20ºC], mixed with wheat flour 50:50, added [salt, sunflower oil, water], rolled and flattened, roasted in oil for 2 min, packed in ziploc bags and stored in -80ºC	Chapati		
			Flour	[Chipped, dried-solar to moisture of 10%, milled, packed in brown sack bags with PE paper and stored at -20ºC], mixed with maize flour 50:50, mixed with water, cooked in hot water for 5 min, cooled, stored at -80ºC	Porridge		
				[Boiled unpeeled 1 hr @100ºC, cooled, pureed by hammer-mill, vacuum packed, stored at -20ºC], mixed with wheat flour 50:50, added [salt, sunflower oil, water], rolled and flattened, roasted in oil for 2 min,	Chapati		

				packed in ziploc bags and stored in -80ºC			
Beauregard Gold II (New)	TCC	HPLC	Flour	[Raw: Peeled, diced, treated with 0.3% metabisulphite solution, dried @40ºC for 48 h with 50 m/min air velocity, milled-hammer into flour], extruded: single-screw volumetric powder feeder Model KX16, Brabender Technologie KG at 30%, 35%, or 40% moisture; or screw speed of 150, 220, 300 rpm, cooled, stored, freeze-dried, cryomilled	Flour, extruded	Australia	[32]

Al, aluminum; AEBC, all-E-beta carotene; ATBC, all-trans beta-carotene; BC, beta-carotene; BCE, BC equivalents; h, hours; HPLC, high-performance liquid chromatography; min, minutes; LLDPE, linear low density polyethylene, NR, not reported; O2, oxygen; OSP, orange sweet potato; PA, polyamide, PE, polyethylene or polythene; PL, polyester; PP, polypropylene; PVA, provitamin A; pVAC, provitamin A content; RH, relative humidity; RP-HPLC, reverse-phase high-performance liquid chromatography; RP-LC, reverse phase liquid chromatography; TCC, total carotenoid content; UV-VIS, ultra-violet visible spectrophotometry
*Country refers to where crops were grown

Supplementary Table 4. Characteristics of included studies: cassava

Varietal	Micro-nutrient	Micronutrient analysis method	Starting product	Processing	Ending product	Country*	Ref
TMS 1358	BC	Absorbance of methanolic extract	Raw, peeled, milled/mashed	Fermented 1–4 days, dewatered, sieved, roasted, stored 0–6 months	Gari	Nigeria	[70]
GM 4571-3<br>GM 5194-13<br>GM 5194-5<br>GM 5194-6<br>GM 4414-5<br>SM 3757-75<br>SM 3758-43<br>SM 3762-15<br>SM 3767-84<br>SM 3774-21<br>SM 3765-15	TCC, BCE	HPLC	Raw, peeled, grated	± Fermented 2 days, oven-dried (40ºC, 2 day), milled, sieved, boiled	Porridge	Colombia	[39]
				± Fermented 2 days, oven-dried (40ºC, 2 day), milled	Flour		
				Fermented 2 days, oven-dried (40ºC, 2 day), milled, sieved	Gari		
				Fermented 2 days, oven-dried (40ºC, 2 day), milled, sieved,, hot water	Eba		
TMS 01/1371<br>TMS 01/1368<br>TMS 01/1371<br>TMS 01/1412	ATBC	HPLC	Raw, peeled	Grated, fermented 3h–3 days, pressed, sifted, roasted, sieved	Gari	Nigeria	[40]
BRS Jari<br>Clone 14-11<br>Clone 03-15<br>IAC 265-97	ATBC	HPLC	Raw, peeled	Boiled	Boiled	Brazil	[71]
				Fried	Fried		

IAC 06-01							
IBA090090<br>IBA090151<br>IBA070557<br>IBA085392<br>IBA083724<br>IBA083774<br>IBA070593<br>IBA070539	TCC	Spectro-photometry	Raw, peeled	Boiled	Boiled	Ghana	72
TMS 0593<br>NR 0220<br>TMS 1371<br>TMS 0593<br>NR 0220<br>TMS 1371	BC	HPLC	Raw, peeled	Grated, fermented 1–4 days, dewatered, sieved, fried	Gari	Nigeria	73
				Grated, fermented 1–4 days, dewatered, sieved, fried, hot water	Eba		
TMS 07-5993<br>TMS 07/0220<br>TMS 01/1371	BC	HPLC	Raw, peeled	Chipped, fermented 4 days, dried (oven 30 min @190 ºC), sun (24 h @ 25 ºC) or flash 5 min @ 180 ºC)	Chips	Nigeria	74
				Fermented 4 days, dried (oven 30 min @190 ºC), sun (24 h @ 25 ºC) or flash 5 min @ 180 ºC), milled, sieved	Flour		
				Fermented 4 days, dried (oven 30 min	Lafun		

				@190 ºC), sun (24 h @ 25 ºC) or flash 5 min @ 180 ºC), milled, sieved, added hot water			
BRS Jari	BC, ATBC	HPLC	Raw, peeled	Boiled	Boiled	Brazil	[75]
				Boiled, fried in soybean oil	Fried		
BRS Dourada<br>BRS Gema de Ovo<br>BRS Jari<br>Hybrid 2003 14-11	TCC	HPLC	Raw, unpeeled	Peeled, sliced, blanched, dehydrated @65ºC, stored 15 days ± onion and parsley flavoring	Chips	Brazil	[76]
MVZ2011B/360<br>MVZ2012/044<br>Kindisa<br>Vuvu	BCE	HPLC	Raw, peeled	Boiled	Boiled		[38]
				Grated, Fermented 3 days, Smashed, Sieved, Pounded, Partially boiled, Kneaded, Steamed (in leaves) 40 min	Chikwangue	Kongo Central	
				Chipped, Fermented 3 days, Dried, milled, boiled	Fufu		
				Chipped, Fermented 3 days, Dried	Microcosettes		
				Grated, Fermented 3 days,	Kimpuka		

				Smashed, Sieved, Pounded			
3 varietals (NR)	BCE	HPLC	Raw, peeled	Peeled, cut, soaked in DI water, boiled 30 min at 95ºC, cooled, mashed, stored at –80ºC	Boiled	USA	[77]
				Peeled, thawed, grated, fermented 3 days, pressed, pulverized, roasted at 195ºC for 20 min or 165 ºC for 5, 10, 15, or 20 min, cooled, pulverized, sieved, then frozen at –80ºC	Gari		
				Peeled, thawed, washed in in DI water, cut, submerged for 5 days at RT, filtered through muslin cloth, fermented, water removed, mixed with fresh water, cooked in pot for 10 min at 100 ºC, cooled for 10 min then frozen at –80ºC	Fufu		

AEBC, all-E-beta carotene; ATBC, all-trans beta-carotene; BC, beta-carotene; BCE, BC equivalents; DAP, days after pollination; h, hours; HPLC, high-performance liquid chromatography; min, minutes; NR, not reported; PVA, provitamin A; pVAC, provitamin A content; RH, relative humidity; TCC, total carotenoid content<br>*Country refers to where crops were grown

Supplementary Table 5. Characteristics of included studies: pearl millet

Varietal	Micro-nutrient	Micronutrient analysis method	Starting product	Processing	Ending product	Country*	Ref
MAHYCO 204<br>ICTP 8203<br>H6M33<br>8203 Nirmal – Mahabeej<br>Mahalakshmi 504	Fe<br>Zn	ICP-OES	Raw, whole	Soaking 12h or 24h using grain:water ratio of 1:2 or 1:5	Soaked	India	41
				Soaking 12h or 24h using grain:water ratio of 1:2 or 1:5 with germination 48 hours	Soaked, germinated		
				Soaking 12h or 24h using grain:water ratio of 1:2 or 1:5 with malting 6 min	Soaked, malted		
Dhanashakti<br>ICMH 1201	Fe<br>Zn	AAS	Raw, whole	Decortication	Decorticated	India	42
				Steeped, fermented 8h, forced draught oven-dried	Steeped, dried		
				Parboiled, forced draught oven-dried	Parboiled, dried		
			Raw, decorticated	Steeped, fermented 8h, decorticated, forced draught oven-dried, air cooled-milled, stored ≤1 month at 10 ºC	Flour		
				Parboiled, decorticated, forced draught oven-dried, aircool-milled, stored ≤1 month @10ºC			
GB8735<br>Tabi	Fe	AAS	Raw, whole	Decorticated	Decortication	Mali	44
AHB 1200 Fe	Fe<br>Zn	ICP-OES	Raw flour	Germinated 72 hrs, oven-dried @ 50ºC	Flour, germinated	India	43

AAS, atomic absorption spectroscopy; Fe, iron; ICP-OES, Inductively coupled plasma atomic emission spectroscopy; h, hours; Zn, zinc
*Country refers to where crops were grown

Supplementary Table 6. Characteristics of included studies: beans

Varietal	Micro-nutrient	Micronutrient analysis method	Starting product	Processing	Ending product	Country*	Ref
Supremo<br>Porto Real<br>Pirata<br>Brazil	Fe<br>Zn	ICP-OES	Raw	Soaked 10 h, added water, autoclaved @121 ºC 10 min, lyophilized, ground, refrigerated	Flour	Brazil	[78]
Combination of BIO 101, BIO 107, ICTA Chortí	Fe<br>Zn	ICP-MS	Raw	Soaked, Boiled	Boiled	Colombia	[45]
				Soaked, Refried	Refried		
				Boiled	Boiled		
				Refried	Refried		
ROBA1<br>K131	Fe<br>Zn	HCl extraction	Raw flour, made by soaking in a grain:water ratio of 1:2 for 24 hours, germinated 48 hours, roasted 170 ºC 45 min, and wonder-milled	Malted/roasted	Malted flour	Uganda	[48]
				Twin screw-extruded from 60ºC to 130ºC to 150ºC, milled	Extruded flour		

Fe, iron; HCl, hypochloric acid; ICP-MS, Inductively coupled plasma mass spectrometry; ICP-OES, Inductively coupled plasma atomic emission spectroscopy; h, hours; min, minutes; Zn, zinc
*Country refers to where crops were grown

Supplementary Table 7. Characteristics of included studies: rice

Varietal	Micro-nutrient	Micronutrient analysis method	Starting product	Processing	Ending product	Country*	Ref
AM1<br>AM143<br>AM180<br>AM65<br>AM72<br>ARB6<br>JEERIGESANNA<br>SEBATI<br>VANDHANA	Fe<br>Zn	ICP-OES	Paddy	Dried to 10% moisture, dehusked	Brown	India	[53]
				Dried to 10% moisture, dehusked, polished to 5% degrees of milling	Polished (5% degrees of milling)		
				Dried to 10% moisture, dehusked, polished to 10% degrees of milling	Polished (10% degrees of milling)		
			Brown	Dried to 10% moisture, dehusked, polished to 5% degrees of milling	Polished (5% degrees of milling)		
			Polished (5%)	Dried to 10% moisture, dehusked, polished to 10% degrees of milling	Polished (10% degrees of milling)		
BRRI dhan64 (BF1)	Fe<br>Zn	ICP-OES	Paddy, sundried to 10–12% moisture)	Steeped/Soaked to reach 33±2% moisture content, Dehulled	Brown rice	Bangladesh	[52]
				Parboiled (Soaked, Steamed 13 min, Immediately cooled), Dried-forced-air oven @30ºC, Dried to 18-22% moisture, Tempered 4-6 hr, 11–13% moisture			
				Parboiled (Soaked, Steamed 16 min, Immediately cooled), Dried-forced-air oven @30ºC, Dried to 18-22% moisture, Tempered 4-6 hr, 11–13% moisture			
				Steeped/Soaked to reach 33±2% moisture content, Dehulled, Polished at 7.5% degrees of milling	Polished (7.5% degrees of milling)		
				Parboiled (Soaked, Steamed 13 min, Gradually cooled to <50 ºC), Dried-forced-air oven @30ºC, Dried to 18-22% moisture, Tempered 4-6 hr, 11–13%			

				moisture, Polished at 7.5% degrees of milling			
				Parboiled (Soaked, Steamed 16 min, Gradually cooled to <50 ºC), Dried-forced-air oven @30ºC, Dried to 18-22% moisture, Tempered 4-6 hr, 11–13% moisture, Polished at 7.5% degrees of milling			
				Steeped/Soaked to reach 33±2% moisture content, Dehulled, Polished at 10% degrees of milling	Polished (10% degrees of milling)		
				Parboiled (Soaked, Steamed 13 min, Gradually cooled to <50 ºC), Dried-forced-air oven @30ºC, Dried to 18-22% moisture, Tempered 4-6 hr, 11–13% moisture, Polished at 10% degrees of milling			
Notes: Fe, iron; ICP-OES, Inductively coupled plasma atomic emission spectroscopy; Zn, zinc<br>*Country refers to where crops were grown							

Supplementary Table 8. Search strategy across included databases			
**Database Name**	**Final Search String**	**Date of search**	**# of records**
MEDLINE	Biofortification[MeSH] OR biofortify*[tiab] OR "bio-fortif*"[tiab]	**2021-03-09**	**1434**
AgEcon	All of the words [biofortify*] in All Fields OR All of the words [bio-fortif*] in All fields	**2021-04-07**	**73**
AGRICOLA	TX (biofortify* OR bio-fortif*) AND TX (Adopt* OR Farmer* OR Household* OR Accept* OR Sensory OR DALY OR "disability adjusted life year*" OR Market* OR School meal program* OR Retention OR Mill* OR Process* OR Stor* OR Cook* OR Polish* OR Bioavailab* OR Cost-effectiveness OR Bioaccessib* OR Bioactiv* OR Efficacy)	**2021-04-07**	**722**
CAB Abstracts	TS=44iofortify* OR TS=bio-fortif* AND TS=(Adopt* OR Farmer* OR Household* OR Accept* OR Sensory OR DALY OR "disability adjusted life year*" OR Market* OR School meal program* OR Retention OR Mill* OR Process* OR Stor* OR Cook* OR Polish* OR Bioavailab* OR Cost-effectiveness OR Bioaccessib* OR Bioactiv* OR Efficacy)	**2021-04-07**	**1538**

Supplementary Table 9. Results from hand-searching organization websites

**Organization website**	**Studies identified on April 7, 2021, and added to screening pool**
HarvestPlus (https://www.harvestplus.org/knowledge-market/publications)	75 (manual)
CIMMYT Publications Repository (https://repository.cimmyt.org/discover)	0 (captured in other databases)
IITA (http://biblio.iita.org/index.php?page=pubyear&kind=year&type=iita) *Currently unavailable	2 (manual)
CIAT (https://ciat.cgiar.org/publications/)	Webpage not working
IRRI (http://scientific-output.irri.org/)	0 (captured in other databases)
ICRISAT (http://oar.icrisat.org/cgi/search/archive/simple?screen= Search&dataset=archive&order=&q=biofortification&_action_search=Search)	151=”biofortify*”
ICARDA (https://www.icarda.org/publications)	0 (irrelevant)
**TOTAL**	**228**

Discussion: Additional points

Micronutrient retention during fresh storage

Only maize and OSP were investigated regarding fresh storage and micronutrient retention, leaving a gap for cassava. PVA degraded by about 50% in raw maize, when stored by as few as 15 days in ambient conditions, and by about 10% in raw OSP stored for 15 days. Pre-conditioning maize kernels at 4 °C may be beneficial prior to freezing at lower temperatures.

Maximizing micronutrient retention after processing

For provitamin A crops, micronutrient retention was maximized when boiling (with a lid) or roasting (in husk) raw maize; drying (using hot air, solar, or crossflow) unpeeled OSP; and boiling whole cassava.

For mineral-biofortified crops, micronutrient retention was highest after parboiling or oven-drying pearl millet; boiling, refrying, or milling beans; dehulling rice without further polishing; and using 95% extraction for milling wheat.

However, we noted several data gaps. For example, we identified only one study examining zinc-biofortified wheat, and there was substantial heterogeneity across the various varietals within each crop, the preparation steps involved, and finished products. Retention varied between the slight differences or steps in preparation methods (for example, fermentation days varying between 0 and 4, days of storage ranging from 0 to 180, or increasing boiling time from 10 to 50 minutes by 10-minute increments), differences in varietals containing different levels of the micronutrient of interest prior to processing, and differences in the starting product (for example, when carotenoids were measured in raw maize kernels aged to different maturities). Regarding provitamin A crop processing, only a few studies described carotenoid degradation kinetics during or after processing. Degradation speed of a given carotenoid depends on the activation energy of the same, which differs by type of carotenoid and processing method including its surrounding medium such as oil or water[79]. For example, the activation energy of beta-cryptoxanthin in oil was found to be higher than that for beta-carotene in a previous study, indicating that a smaller temperature change is needed to degrade beta-carotene more rapidly in an oil medium[79]. Particular crop varieties and subsequently, the baseline level of micronutrient(s) prior to processing also varied substantially across crops and impacted the final amount of micronutrient retained. Therefore, as new varieties of biofortified crops are released to farmers and markets, it appears critical to know the exact varietal of each crop and the baseline micronutrient content range in order to know which type of processing method to use to maximize micronutrient retention.

Maximizing micronutrient retention during storage of processed crops

Micronutrient retention in crops after processing and storage were explored in maize, OSP, and pearl millet. PVA content in cooked maize food products such as porridge appeared robust for up to 90 days, while storing whole cooked kernels or cobs required specific packaging such as aluminium bags with oxygen scavengers for several months' storage. Oxygen transmission into packaging during storage causes greater loss of beta carotene due to degradation[37]. Similarly, PVA retention was maximized in OSP flour by using packaging materials that prevent

degradation and by deep freezing cooked OSP. Refrigerating pearl millet for ≤1 month did not negatively impact iron or zinc retention.

Coverage of common processing methods

Micronutrient retention values are only useful to the home user if they represent processes that would be commonly used. Common processing methods across the crops were fairly well represented by the literature base for OSP, cassava, pearl millet, beans, and wheat, but missing processing methods were found for other crops. In Nigeria, two common maize processing methods were not found in the literature: kokoro, a dough that is then cut into shapes and fried; and eko or agidi, the result of processing maize into a stiff porridge that is then leaf-wrapped and allowed to cool *(personal communication)*. A common Tanzanian meal, makande, which is boiled maize that is then mixed with beans *(personal communication)*, was also not described in terms of micronutrient retention. In Bangladesh, rice is often consumed in a flattened or puffed form *(personal communication)*, neither of which were found in our review. It is important to elucidate the micronutrient retention capacity of these common processing methods if these are the primary ways in which a particular crop is actually consumed.

Varietal-related differences

Aside from baseline micronutrient content, which also varied by biofortified crop, differences in the physical and biochemical makeup of each crop varietal or genotype may contribute to the differences in micronutrient retention after processing.

Biofortified maize genotypes differ in kernel characteristics, such as the how flint or dent (hard or soft) the endosperm is, as well as starch granule and protein body organization. Kernel hardness is associated with the ability to store different carotenoids; as one example, a previous study found that hard endosperm genotypes had more provitamin A compared to soft, starchy endosperm genotypes, and flint genotypes had higher beta-branch carotenoids including BC compared to dented genotypes [80]. Further, variation in *ZEP* gene expression during seed development has been shown to impact carotenoid content[81], as well as stability, in maize seeds, which also may be impacted by maturation stage, e.g., milky stage compared to dough stage, indicated by kernel moisture percentage[9].

OSP varieties/genotypes may differ in root size, and even within one genotype root size was positively associated with BC content in a previous study[28], in addition to color, water content, dry matter content, and susceptibility to viruses[82]. Beta-carotene content was higher in OSP cultivated at high altitudes and at semi-arid highlands compared to low altitudes and arid lowlands[82]. In terms of retention, one study showed that greater dry matter content was associated with lower carotenoid losses[69]; dry matter content may be impacted by the weather, soil, and farming practices during the growing season as well as maturity[83].

Cassava genotypes vary by dry matter content, in addition to pest resistance, mealiness, beta-carotene and starch content. In one study, varieties that had a higher baseline ATBC content retained less ATBC than varieties with lower ATBC, after boiling[71]. Studies on how characteristics of different genotypes relate to PVA retention are lacking.

Biofortified pearl millet varieties can vary in physical characteristics, such as having greater kernel weight, size, and hardness, as well as chemical composition such as higher protein and

fat and lower crude fiber compared to non-biofortified pearl millet[42]. Antinutrients, such as phytates and phenols, may be higher in the biofortified genotypes[42]. Kernel weight, size, and fat content were positively correlated with iron content in one study[42].

In beans, targets for increased mineral concentration include the seed coat, cotyledons, and embryo[84]; it is also important to account for phytic acid in the cotyledons and polyphenols in the seed coat[85]. Bean seed size, and to a lesser extent seed coat thickness may vary by genotype[84]. Seed coat determines the beans' ability to absorb water which translates to differences in cooking time; a thinner seed coat results in reduced cooking time[86].

Iron and zinc vary in content and are distributed in different parts of the rice grain, depending on genotype[87]. Taller varieties may have larger kernel sizes that result in greater nutrient content[87]. Iron and zinc location in the rice grain is also key to retention especially during necessary processes such as dehusking as well as quality-enhancing processing such as polishing. While zinc transporters in the husk may translocate zinc into the endosperm, it appears iron transporters are less efficient in this process, resulting in iron—but not zinc—losses during dehusking[87]. The same study showed that micronutrients appeared to be concentrated in the outer aleurone layer of the grain, given that iron and zinc losses were greatest during the first level of polishing but not in subsequent deeper polishing[87]. Thus, genotypes that allow greater transport of iron and zinc to the endosperm would maintain higher levels of mineral retention.

Apparent retention vs. true retention

As opposed to apparent retention, which is measured on a dry weight basis, true retention is measured on a fresh weight basis. This improves the method's accuracy by accounting for water and solid losses or gains. True retention can be used for the maize flour above if not much mass is loss. Since in many processes a lot of mass is loss (milling, sieving, soaking, etc.,), users of true retention values need to be careful when estimating nutrient content of final products using the nutrient concentration of the harvested crop. Generally, the users that want to estimate the nutrient concentration in the final product are not interested in the amount of mass loss, so, they are not interested in TR.

AR holds the assumption that weight changes—such as water and other solid losses when vegetables are steamed or grains are milled, or gains, such as when rice is cooked or oil is added—are negligible. Therefore, apparent retention can be used to describe food processing where solid losses are negligible (e.g., drying), but it may not be accurate when discussing other food processing methods such as, milling, cooking, or blanching[1, 2]. When trying to estimate the nutrient concentration of a product (e.g., maize flour) at the end of its shelf life for nutrient labelling purposes, a reference of the initial concentration of the nutrient in the harvested crop and the expected concentration in the flour at the end of its shelf life is needed. Having an AR for such a process can be useful to elaborate the nutrient labelling. Determining/having the true retention TR is not necessary in this case. If only true retention is available for the example above (and not AR available), the yield of the milling process is needed to properly estimate the values to use for labelling. Therefore, true retention is not always better than apparent retention.

Supplementary Information References

1. Murphy EW, Criner PE, Gray BC. Comparisons of methods for calculating retentions of nutrients in cooked foods. *Journal of Agricultural and Food Chemistry* **23**, 1153-1157 (1975).

2. Bechoff A, Taleon V, Carvalho LMJ, Carvalho JLV, Boy E. Micronutrient (provitamin A and iron/zinc) retention in biofortified crops. *African Journal of Food, Agriculture, Nutrition and Development* **17**, 11893-11904 (2017).

3. Bechoff A, Tomlins KI, Dhuique-Mayer C, Westby A. Understanding carotenoid losses in orange-fleshed sweet potato in drying and storage. *Tropical roots and tubers in a changing climate: a convenient opportunity for the world Fifteenth Triennial Symposium of the International Society for Tropical Root Crops, Lima, Peru, 2-6 November 2009*, 70-77 (2009).

4. Mugode L*, et al.* Carotenoid retention of biofortified provitamin A maize (Zea mays L.) after Zambian traditional methods of milling, cooking and storage. *J Agric Food Chem* **62**, 6317-6325 (2014).

5. Md Saleh R, Kulig B, Hensel O, Sturm B. Investigation of dynamic quality changes and optimization of drying parameters of carrots (Daucus carota var. laguna). *Journal of Food Process Engineering* **43**, e13314 (2020).

6. Calvo-Brenes P, O'Hare T. Effect of freezing and cool storage on carotenoid content and quality of zeaxanthin-biofortified and standard yellow sweet-corn (Zea mays L.). *Journal of Food Composition and Analysis* **86**, 103353 (2020).

7. Barbosa NA, Paes MCD, Guimaraes PEdO, Pereira J. Carotenoid retention in minimally processed biofortified green corn stored under retail marketing conditions. *Ciencia e Agrotecnologia* **39**, 363-371 (2015).

8. Barbosa NA, Paes MCD, Guimaraes PEdO, Pereira J. Carotenoid retention in immature corn ear grains subjected to different thermal treatments. *Journal of Agricultural Science (Toronto)* **7**, 177-186 (2015).

9. Cabrera-Soto L, Aldo R-N, Kevin VP, Luis AG-F, Natalia P-R. Carotenoid and Tocochromanol Profiles during Kernel Development Make Consumption of Biofortified “Fresh” Maize an Option to Improve Micronutrient Nutrition. *Journal of agricultural and food chemistry* **66**, 9391-9398 (2018).

10. Rosales A, Agama-Acevedo E, Arturo Bello-PeÌ□rez L, GutieÌ□rrez-Dorado R, Palacios-Rojas N. Effect of Traditional and Extrusion Nixtamalization on Carotenoid Retention in Tortillas Made from Provitamin A Biofortified Maize (Zea mays L.). *Journal of agricultural and food chemistry* **64**, 8289-8295 (2016).

11. Pillay K, Siwela M, Derera J, Veldman FJ. Provitamin A carotenoids in biofortified maize and their retention during processing and preparation of South African maize foods. *J Food Sci Technol* **51**, 634-644 (2014).

12. Ortiz D, Nkhata SG, Rocheford T, Ferruzzi MG. Steeping of Biofortified Orange Maize Genotypes for Ogi Production Modifies Pasting Properties and Carotenoid Stability. *Agronomy* **9**, (2019).

13. Beswa D, Siwela M, Amonsou EO, Kolanisi U. Grain Quality, Provitamin A Carotenoid Profiles, and Sensory Quality of Provitamin A-Biofortified Maize Stiff Porridges. *Foods* **9**, (2020).

14. Nkhata SG, Ortiz D, Baributsa D, Hamaker B, Rocheford T, Ferruzzi MG. Assessment of oxygen sequestration on effectiveness of Purdue Improved Crop Storage (PICS) bags in reducing carotenoid degradation during post-harvest storage of two biofortified orange maize genotypes. *Journal of Cereal Science* **87**, 68-77 (2019).

15. Taleon V, Mugode L, Cabrera-Soto L, Palacios-Rojas N. Carotenoid retention in biofortified maize using different post-harvest storage and packaging methods. *Food Chem* **232**, 60-66 (2017).

16. Ekpa O, Fogliano V, Linnemann A. Carotenoid stability and aroma retention during the post-harvest storage of biofortified maize. *J Sci Food Agric*, (2020).

17. Ortiz D, Rocheford T, Ferruzzi MG. Influence of Temperature and Humidity on the Stability of Carotenoids in Biofortified Maize (Zea mays L.) Genotypes during Controlled Postharvest Storage. *J Agric Food Chem* **64**, 2727-2736 (2016).

18. Kramer A. Effect of storage on the nutritive value of food. *Journal of Food Quality* **1**, 23-55 (1977).

19. Pereira EJ*, et al.* Effects of cooking methods on the iron and zinc contents in cowpea (Vigna unguiculata) to combat nutritional deficiencies in Brazil. *Food Nutr Res* **58**, (2014).

20. Mitra S, Tarafdar J, Palaniswami MS. Impacts of different maturity stages and storage on nutritional changes in raw and cooked tubers of orange-fleshed sweet potato (Ipomoea batatas) cultivars. In: *Acta Horticulturae* (ed Herppich WB) (2010).

21. Bengtsson A, Namutebi A, Alminger M, Svanberg U. Effects of various traditional processing methods on the all-trans0≤-carotene content of orange-fleshed sweet potato. *Journal of Food Composition and Analysis* **21**, 134-143 (2008).

22. Bechoff A, Dufour D, Dhuique-Mayer C, Marouzé C, Reynes M, Westby A. Effect of hot air, solar and sun drying treatments on provitamin A retention in orange-fleshed sweetpotato. *Journal of Food Engineering* **92**, 164-171 (2009).

23. Bechoff A*, et al.* Effect of drying and storage on the degradation of total carotenoids in orange-fleshed sweetpotato cultivars. *Journal of the Science of Food and Agriculture* **90**, 622-629 (2010).

24. Vimala B, Nambisan B, Hariprakash B. Retention of carotenoids in orange-fleshed sweet potato during processing. *J Food Sci Technol* **48**, 520-524 (2011).

25. Donado-Pestana CM, Mastrodi Salgado J, de Oliveira Rios A, dos Santos PR, Jablonski A. Stability of carotenoids, total phenolics and in vitro antioxidant capacity in the thermal processing of orange-fleshed sweet potato (Ipomoea batatas Lam.) cultivars grown in Brazil. *Plant Foods Hum Nutr* **67**, 262-270 (2012).

26. Tiruneh Y, Urga K, Bekerie A, Tassew G. Effect of Treatment on the Beta Carotene Retention of Orange Fleshed Sweet Potato Varieties Grown in Hawassa, Ethiopia. *Journal of Nutrition and Food Sciences* **11**, 1-4 (2021).

27. Failla ML, Thakkar SK, Kim JY. In vitro bioaccessibility of beta-carotene in orange fleshed sweet potato (Ipomoea batatas, Lam.). *J Agric Food Chem* **57**, 10922-10927 (2009).

28. Wu X, Sun C, Yang L, Zeng G, Liu Z, Li Y. β-carotene content in sweet potato varieties from China and the effect of preparation on β-carotene retention in the Yanshu No. 5. *Innovative Food Science & Emerging Technologies* **9**, 581-586 (2008).

29. Kidmose U, Christensen LP, Agili SM, Thilsted SH. Effect of home preparation practices on the content of provitamin A carotenoids in coloured sweet potato varieties (Ipomoea batatas Lam.) from Kenya. *Innovative Food Science & Emerging Technologies* **8**, 399-406 (2007).

30. Islam SN, Nusrat T, Begum P, Ahsan M. Carotenoids and β-carotene in orange fleshed sweet potato: A possible solution to vitamin A deficiency. *Food Chemistry* **199**, 628-631 (2016).

31. K'Osambo LM, Carey EE, Misra AK, Wilkes J, Hagenimana V. Influence of Age, Farming Site, and Boiling on Pro-Vitamin A Content in Sweet Potato (Ipomoea batatas(L.) Lam.) Storage Roots. *Journal of Food Composition and Analysis* **11**, 305-321 (1998).

32. Waramboi JG, Gidley MJ, Sopade PA. Carotenoid contents of extruded and non-extruded sweetpotato flours from Papua New Guinea and Australia. *Food Chemistry* **141**, 1740-1746 (2013).

33. Bechoff A*, et al.* Retention and bioaccessibility of beta-carotene in blended foods containing orange-fleshed sweet potato flour. *J Agric Food Chem* **59**, 10373-10380 (2011).

34. Chilungo S, Muzhingi T, Truong V-D, Allen JC. Effect of processing and oil type on carotene bioaccessibility in traditional foods prepared with flour and puree from orange-fleshed sweetpotatoes. *International Journal of Food Science & Technology* **54**, 2055-2063 (2019).

35. Alves RMGe, Ito D, Carvalho JLV, Melo WFd, Godoy RLO. Estabilidade de farinha de batata-doce biofortificada Stability of biofortified sweet potato flour. *Brazilian Journal of Food Technology* **15**, 59-71 (2012).

36. Bechoff A*, et al.* Relationship between the kinetics of β-carotene degradation and formation of norisoprenoids in the storage of dried sweet potato chips. *Food Chemistry* **121**, 348-357 (2010).

37. Emenhiser C*, et al.* PACKAGING PRESERVATION OF β-CAROTENE IN SWEET POTATO FLAKES USING FLEXIBLE FILM AND AN OXYGEN ABSORBER. *Journal of Food Quality* **22**, 63-73 (1999).

38. Taleon V, Sumbu D, Muzhingi T, Bidiaka S. Carotenoids retention in biofortified yellow cassava processed with traditional African methods. *J Sci Food Agric* **99**, 1434-1441 (2019).

39. Aragón IJ, Ceballos H, Dufour D, Ferruzzi MG. Pro-vitamin A carotenoids stability and bioaccessibility from elite selection of biofortified cassava roots (Manihot esculenta, Crantz) processed to traditional flours and porridges. *Food Funct* **9**, 4822-4835 (2018).

40. Bechoff A, Tomlins KI, Chijioke U, Ilona P, Westby A, Boy E. Physical losses could partially explain modest carotenoid retention in dried food products from biofortified cassava. *PLoS One* **13**, e0194402 (2018).

41. Thorat A*, et al.* Effect of Processing on Total Iron and Zinc, Ionizable Iron, Extractable Zinc, Phytate and Phytate: Mineral Ratios in Pearl Millet. *Advances in Food Science and Engineering* **1**, 129-143 (2017).

42. Gwamba J, Kruger J, Taylor JRN. Influence of grain quality characteristics and basic processing technologies on the mineral and antinutrient contents of iron and zinc biofortified open-pollinated variety and hybrid-type pearl millet. *International journal of food science & technology* **55**, 1547-1558 (2020).

43. Kale PG, Babar KP, Bornare DT, Vairagar PR. Evaluation of physical, nutritional and sensory characteristics of cookies developed with bio-fortified pearl millet. *Food Science Research Journal* **9**, 223-230 (2018).

44. Hama-Ba F, Mouquet-Rivier C, Diawara B, Weltzien E, Icard-Vernière C. Traditional African Dishes Prepared From Local Biofortified Varieties of Pearl Millet: Acceptability and Potential Contribution to Iron and Zinc Intakes of Burkinabe Young Children. *Front Nutr* **6**, 115 (2019).

45. Hummel M*, et al.* Iron, Zinc and Phytic Acid Retention of Biofortified, Low Phytic Acid, and Conventional Bean Varieties When Preparing Common Household Recipes. *Nutrients* **12**, (2020).

46. Brigide P, Canniatt-Brazaca SG, Silva MO. Nutritional characteristics of biofortified common beans. *Food Science and Technology (Campinas)* **34**, 493-500 (2014).

47. Feitosa S, Greiner R, Meinhardt AK, Muller A, Almeida DT, Posten C. Effect of Traditional Household Processes on Iron, Zinc and Copper Bioaccessibility in Black Bean (Phaseolus vulgaris L.). *Foods* **7**, (2018).

48. Nkundabombi MG, Nakimbugwe D, Muyonga JH. Effect of processing methods on nutritional, sensory, and physicochemical characteristics of biofortified bean flour. *Food Sci Nutr* **4**, 384-397 (2016).

49. Marzo F, Alonso R, Urdaneta E, Arricibita FJ, Ibanez F. Nutritional quality of extruded kidney bean (Phaseolus vulgaris L. var. Pinto) and its effects on growth and skeletal muscle nitrogen fractions in rats. *J Anim Sci* **80**, 875-879 (2002).

50. Audu S, Aremu M. Effect of Processing on Chemical Composition of Red Kidney Bean (Phaseolus vulgaris L.) Flour. *Pakistan Journal of Nutrition* **10**, (2011).

51. Edwards RH, Becker R, Mossman AP, Gray GM, Whitehand LC. Twin-Screw Extrusion Cooking of Small White Beans (Phaseolus vulgaris). *LWT - Food Science and Technology* **27**, 472-481 (1994).

52. Taleon V, Gallego S, Orozco JC, Grenier C. Retention of Zn, Fe and phytic acid in parboiled biofortified and non-biofortified rice. *Food Chem X* **8**, 100105 (2020).

53. Khan JA, Halagappa Eshwarappa S, Krupa Kuntanahalli N, Sowjanya Makkivalli S, Sumantha H, Zahoor AD. Micronutrient productivity: a comprehensive parameter for biofortification in rice (Oryza sativa L.) grain. *Journal of the science of food and agriculture* **99**, 1311-1321 (2019).

54. Rosado JL*, et al.* The quantity of zinc absorbed from wheat in adult women is enhanced by biofortification. *J Nutr* **139**, 1920-1925 (2009).

55. Awobusuyi TD, Siwela M, Kolanisi U, Amonsou EO. Provitamin A retention and sensory acceptability of amahewu, a non-alcoholic cereal-based beverage made with provitamin A-biofortified maize. *J Sci Food Agric* **96**, 1356-1361 (2016).

56. Alamu EO, Maziya-Dixon B, Menkir A, Ogunlade AO, Olaofe O. Harvesting time and roasting effects on colour properties, xanthophylls, phytates, tannins and vitamin C contents of orange maize hybrid. *Sci Rep* **10**, 21327 (2020).

57. Calvo-Brenes P, Fanning K, O'Hare T. Does kernel position on the cob affect zeaxanthin, lutein and total carotenoid contents or quality parameters, in zeaxanthin-biofortified sweet-corn? *Food Chem* **277**, 490-495 (2019).

58. Dube N, Dinesh Kumar B, Firoz H, Longvah T, Purna Chandra M, Raghu P. β-Carotene bioaccessibility from biofortified maize (Zea mays) is related to its density and is negatively influenced by lutein and zeaxanthin. *Food & function* **9**, 379-388 (2018).

59. Gannon BM, Pixley KV, Tanumihardjo SA. Maize Milling Method Affects Growth and Zinc Status but Not Provitamin A Carotenoid Bioefficacy in Male Mongolian Gerbils. *J Nutr* **147**, 337-345 (2017).

60. Li S, Tayie FA, Young MF, Rocheford T, White WS. Retention of provitamin A carotenoids in high beta-carotene maize (Zea mays) during traditional African household processing. *J Agric Food Chem* **55**, 10744-10750 (2007).

61. Ortiz D, Amudhan P, Bonnet JP, Rocheford T, Ferruzzi MG. Carotenoid stability during dry milling, storage, and extrusion processing of biofortified maize genotypes. *Journal of Agricultural and Food Chemistry* **66**, 4683-4691 (2018).

62. Sowa M*, et al.* Retention of Carotenoids in Biofortified Maize Flour and β-Cryptoxanthin-Enhanced Eggs after Household Cooking. *ACS Omega* **2**, 7320-7328 (2017).

63. Bechoff AW, Andrew; Dufour, Dominique L.; Dhuique-Mayer, C.; Marouzé, C.; Owori, C.; Menya, G.; Tomlins, K.I. Effect of drying and storage on the content of provitamin A of orange fleshed sweet potato (Ipomoa batatas): direct sun radiations do not have significant impact [poster] [on line]. *AgroSalud*, 1 p (2007).

64. Bechoff A, Tomlins K, Dhuique-Mayer C, Dove R, Westby A. On-farm evaluation of the impact of drying and storage on the carotenoid content of orange-fleshed sweet potato (Ipomea batata Lam.). *International Journal of Food Science & Technology* **46**, 52-60 (2011).

65. Bechoff A, Westby A, Menya G, Tomlins KI. EFFECT OF PRETREATMENTS FOR RETAINING TOTAL CAROTENOIDS IN DRIED AND STORED ORANGE-FLESHED-SWEET POTATO CHIPS. *Journal of Food Quality* **34**, 259-267 (2011).

66. Marangoni Junior L, Ito D, Ribeiro SML, Silva MGd, Alves RMV. Stability of beta-carotene rich sweet potato chips packed in different packaging systems. *LWT - Food Science and Technology* **92**, 442-450 (2018).

67. Nherera-Chokuda FV, Smit CJ, Muya MC, Marumo JL. Effect of heat treatment on forage quality of bio-fortified orange fleshed Ipomea batatas crop residues and roots. *African Journal of Agricultural Research* **12**, 2499-2506 (2017).

68. Kidmose U, Yang RY, Thilsted SH, Christensen LP, Brandt K. Content of carotenoids in commonly consumed Asian vegetables and stability and extractability during frying. *Journal of Food Composition and Analysis* **19**, 562-571 (2006).

69. Hagenimana V, Carey EE, Gichuki ST, Oyunga MA, Imungi JK. Carotenoid contents in fresh, dried and processed sweetpotato products. *Ecology of Food and Nutrition* **37**, 455-473 (1998).

70. Abiodun OA, Ayano B, Amanyunose AA. Effect of fermentation periods and storage on the chemical and physicochemical properties of biofortified cassava gari. *Journal of Food Processing and Preservation* **44**, (2020).

71. Berni P, Chitchumroonchokchai C, Canniatti-Brazaca SG, De Moura FF, Failla ML. Impact of genotype and cooking style on the content, retention, and bioacessibility of β-carotene in biofortified cassava (Manihot esculenta Crantz) conventionally bred in Brazil. *J Agric Food Chem* **62**, 6677-6686 (2014).

72. Boakye Peprah B, Parkes EY, Harrison OA, van Biljon A, Steiner-Asiedu M, Labuschagne MT. Proximate Composition, Cyanide Content, and Carotenoid Retention after Boiling of Provitamin A-Rich Cassava Grown in Ghana. *Foods* **9**, (2020).

73. Eyinla TE, Maziya-Dixon B, Alamu OE, Sanusi RA. Retention of Pro-Vitamin A Content in Products from New Biofortified Cassava Varieties. *Foods* **8**, (2019).

74. Eyinla T, Sanusi R, Alamu E, Maziya-Dixon B. Variations of beta-carotene retention in a staple produced from yellow fleshed cassava roots through different drying methods. *Functional Foods in Health and Disease* **8**, 372-384 (2018).

75. Gomes S, Torres AG, Godoy R, Pacheco S, Carvalho J, Nutti M. Effects of boiling and frying on the bioaccessibility of beta-carotene in yellow-fleshed cassava roots (Manihot esculenta Crantz cv. BRS Jari). *Food Nutr Bull* **34**, 65-74 (2013).

76. Oliveira LAd, Reis RC, Santana HM, Santos VdS, Carvalho JLVd. Development and sensorial acceptance of biofortified dehydrated cassava chips. *Semina: Ciencias Agrarias (Londrina)* **38**, 3579-3590 (2017).

77. Thakkar SK, Huo T, Maziya-Dixon B, Failla ML. Impact of Style of Processing on Retention and Bioaccessibility of β-Carotene in Cassava (Manihot esculanta, Crantz). *Journal of Agricultural and Food Chemistry* **57**, 1344-1348 (2009).

78. Brigide P, de Carvalho RV, de Toledo NMV, Frontela Saseta C, López-Nicolás Rn, Ros G. Fe and Zn in vitro bioavailability in relation to antinutritional factors in biofortified beans subjected to different processes. *Food & function* **10**, 4802-4810 (2019).

79. Valerio PP, Frias JM, Cren EC. Thermal degradation kinetics of carotenoids: Acrocomia aculeata oil in the context of nutraceutical food and bioprocess technology. *Journal of Thermal Analysis and Calorimetry* **143**, 2983-2994 (2021).

80. Saenz E, Borrás L, Gerde JA. Carotenoid profiles in maize genotypes with contrasting kernel hardness. *Journal of Cereal Science* **99**, 103206 (2021).

81. Vallabhaneni R, Wurtzel ET. Timing and biosynthetic potential for carotenoid accumulation in genetically diverse germplasm of maize. *Plant Physiol* **150**, 562-572 (2009).

82. Lamaro GP, Tsehaye Y, Girma A, Vannini A, Fedeli R, Loppi S. Evaluation of Yield and Nutraceutical Traits of Orange-Fleshed Sweet Potato Storage Roots in Two Agro-Climatic Zones of Northern Ethiopia. *Plants (Basel)* **12**, (2023).

83. Tomlins K, Owori C, Bechoff A, Menya G, Westby A. Relationship among the carotenoid content, dry matter content and sensory attributes of sweet potato. *Food Chemistry* **131**, 14-21 (2012).

84. Blair MW, Izquierdo P, Astudillo C, Grusak MA. A legume biofortification quandary: variability and genetic control of seed coat micronutrient accumulation in common beans. *Front Plant Sci* **4**, 275 (2013).

85. Sperotto RA, Ricachenevsky FK. Common Bean Fe Biofortification Using Model Species' Lessons. *Front Plant Sci* **8**, 2187 (2017).

86. Santos GGd, Ribeiro ND, Maziero SM. Evaluation of common bean morphological traits identifies grain thickness directly correlated with cooking time. *Pesquisa Agropecuária Tropical* **46**, (2016).

87. Khan AM, Shahid H, Rengel Z, Shah MAA. Zinc bioavailability and nitrogen concentration in grains of wheat crop sprayed with zinc sulfate, ammonium sulfate, ammonium chloride, and urea. *Journal of Plant Nutrition* **41**, 1926-1936 (2018).
